# Rates of Brain Atrophy Across Disease Stages in Familial Frontotemporal Dementia Associated With *MAPT*, *GRN*, and *C9orf72* Pathogenic Variants

**DOI:** 10.1001/jamanetworkopen.2020.22847

**Published:** 2020-10-28

**Authors:** Adam M. Staffaroni, Sheng-Yang M. Goh, Yann Cobigo, Elise Ong, Suzee E. Lee, Kaitlin B. Casaletto, Amy Wolf, Leah K. Forsberg, Nupur Ghoshal, Neill R. Graff-Radford, Murray Grossman, Hilary W. Heuer, Ging-Yuek R. Hsiung, Kejal Kantarci, David S. Knopman, Walter K. Kremers, Ian R. Mackenzie, Bruce L. Miller, Otto Pedraza, Katya Rascovsky, M. Carmela Tartaglia, Zbigniew K. Wszolek, Joel H. Kramer, John Kornak, Bradley F. Boeve, Adam L. Boxer, Howard J. Rosen

**Affiliations:** 1 Department of Neurology, Memory and Aging Center, Weill Institute for Neurosciences, University of California, San Francisco; 2 Department of Neurology, College of Medicine, Mayo Clinic, Rochester, Minnesota; 3 Department of Neurology, Washington University School of Medicine in St Louis, St Louis, Missouri; 4 Department of Psychiatry, Washington University School of Medicine in St Louis, St Louis, Missouri; 5 Department of Neurology, Mayo Clinic, Jacksonville, Florida; 6 Department of Neurology, Perelman School of Medicine, University of Pennsylvania, Philadelphia; 7 Division of Neurology, Department of Medicine, University of British Columbia, Vancouver, British Columbia, Canada; 8 Department of Radiology, College of Medicine, Mayo Clinic, Rochester, Minnesota; 9 Department of Health Sciences Research, Mayo Clinic, Rochester, Minnesota; 10 Department of Pathology and Laboratory Medicine, University of British Columbia, Vancouver, British Columbia, Canada; 11 Department of Psychiatry and Psychology, Mayo Clinic, Jacksonville, Florida; 12 Division of Neurology, Department of Medicine, Tanz Centre for Research in Neurodegenerative Diseases, University of Toronto, Toronto, Ontario, Canada; 13 Department of Epidemiology and Biostatistics, Memory and Aging Center, University of California, San Francisco

## Abstract

**Importance:**

Several clinical trials are planned for familial forms of frontotemporal lobar degeneration (f-FTLD). Precise modeling of brain atrophy in f-FTLD could improve the power to detect a treatment effect.

**Objective:**

To characterize regions and rates of atrophy in the 3 primary f-FTLD genetic groups (*MAPT*, *GRN*, and *C9orf72*) across all disease stages from asymptomatic to dementia.

**Design, Setting, and Participants:**

This investigation was a case-control study of participants enrolled in the Advancing Research and Treatment for Frontotemporal Lobar Degeneration or Longitudinal Evaluation of Familial Frontotemporal Dementia studies. The study took place at 18 North American academic medical centers between January 2009 and September 2018. Participants with f-FTLD (n = 100) with a known pathogenic variant (*MAPT* [n = 28], *GRN* [n = 33], or *C9orf72* [n = 39]) were grouped according to disease stage (ie, Clinical Dementia Rating [CDR] plus National Alzheimer’s Coordinating Center [NACC] FTLD module). Included were participants with at least 2 structural magnetic resonance images at presymptomatic (CDR + NACC FTLD = 0 [n = 57]), mild or questionable (CDR + NACC FTLD = 0.5 [n = 15]), or symptomatic (CDR + NACC FTLD = ≥1 [n = 28]) disease stages. The control group included family members of known pathogenic variant carriers who did not carry the pathogenic variant (n = 60).

**Main Outcomes and Measures:**

This study fitted bayesian linear mixed-effects models in each voxel of the brain to quantify the rate of atrophy in each of the 3 genes, at each of the 3 disease stages, compared with controls. The study also analyzed rates of clinical decline in each of these groups, as measured by the CDR + NACC FTLD box score.

**Results:**

The sample included 100 participants with f-FTLD with a known pathogenic variant (mean [SD] age, 50.48 [13.78] years; 53 [53%] female) and 60 family members of known pathogenic variant carriers who did not carry the pathogenic variant (mean [SD] age, 47.51 [12.43] years; 36 [60%] female). *MAPT* and *GRN* pathogenic variants were associated with increased rates of volume loss compared with controls at all stages of disease. In *MAPT* pathogenic variant carriers, statistically significant regions of accelerated volume loss compared with controls were identified in temporal regions bilaterally in the presymptomatic stage, with global spread in the symptomatic stage. For example, mean [SD] rates of atrophy in the left temporal were −231 [47] mm^3^ per year during the presymptomatic stage, −381 [208] mm^3^ per year during the mild stage, and −1485 [1025] mm^3 ^per year during the symptomatic stage (*P* < .05). *GRN* pathogenic variant carriers generally had minimal increases in atrophy rates between the presymptomatic and mild stages, with rapid increases in atrophy rates in the symptomatic stages. For example, in the right frontal lobes, annualized volume loss was −267 [81] mm^3^ per year in the presymptomatic stage and −182 [90] mm^3 ^per year in the mild stage, but −1169 [555] mm^3^ per year in the symptomatic stage. Compared with the other groups, *C9orf72* expansion carriers showed minimal increases in rate of volume loss with disease progression. For example, the mean (SD) annualized rates of atrophy in the right frontal lobe in *C9orf72* expansion carriers was −272 (118) mm^3^ per year in presymptomatic stages, −310 (189) mm^3^ per year in mildly symptomatic stages, and −251 (145) mm^3^ per year in symptomatic stages.

**Conclusions and Relevance:**

These findings are relevant to clinical trial planning and suggest that the mechanism by which *C9orf72* pathogenic variants lead to symptoms may be fundamentally different from the mechanisms associated with other pathogenic variants.

## Key Points

**Question:**

How does the trajectory of atrophy differ between the 3 primary genetic groups (*MAPT*, *GRN*, and *C9orf72*) associated with familial frontotemporal lobar degeneration?

**Findings:**

Among 160 members of families affected by familial frontotemporal lobar degeneration in this case-control study, *MAPT* and *GRN* pathogenic variants were associated with increases in the rate of volume loss as a function of disease stage, whereas *C9orf72* expansion carriers showed minimal increases in the rate of volume loss with disease progression.

**Meaning:**

This study advances the knowledge of between-gene differences in atrophy rates as a function of disease severity; treatment studies enrolling familial frontotemporal dementia cases should consider the heterogeneity conferred by both the altered gene and the disease stage.

## Teaser

This observation study characterizes regions and rates of atrophy in the 3 primary familial frontotemporal lobar degeneration genes (*MAPT*, *GRN*, and *C9orf72*) across all disease stages from asymptomatic to dementia.

## Introduction

Frontotemporal lobar degeneration (FTLD) is a neurodegenerative disorder associated with a variety of pathological mechanisms. As many as 30% of FTLD cases are associated with pathogenic gene variants that are autosomal dominant (familial forms of FTLD [f-FTLD]), and over half of these are associated with pathogenic variants in 1 of the following 3 genes: microtubule-associated protein tau (*MAPT* [OMIM 157140]), progranulin (*GRN* [OMIM 138945]), and a repeat expansion in the chromosome 9 open reading frame 72 (*C9orf72* [OMIM 614260]) gene. Pathogenic variants in each of these genes are associated with overlapping but unique clinical and neuroimaging manifestations.^1,2,3,4,5,6,7,8^

Accurate characterization of the natural history of each genetic group is important for clinical care and clinical trials because precise modeling of the disease course can improve the ability to detect a treatment effect.^9,10^ Furthermore, there is a need for a working model of disease and biomarker progression in f-FTLD to inform hypotheses about when biomarker changes develop in the course of disease and how biomarkers change over time.^11^ In addition, natural history data can help clinicians prognosticate and assist family planning.

Many studies have used brain atrophy to describe the evolution of neurodegeneration in f-FTLD, yielding the following observations: (1) cross-sectional atrophy can be detected in the presymptomatic stages, and each genetic group has different regional predilection for atrophy^3,6,8,12,13,14^; (2) atrophy rates in the presymptomatic stages may exceed those of age-matched control cases^5,15,16^; (3) the rate of volume loss may accelerate near the transition from asymptomatic to symptomatic^5,13^; and (4) volume loss in symptomatic cases is usually well in excess of that in control cases.^16,17,18^ However, conclusions from these observations are tempered because many analyses focused only on 1 genetic group or disease stage, limiting comparisons across genes and stages. Moreover, many prior estimates of change over time were derived from cross-sectional rather than longitudinal data.

The emergence of large, comprehensive studies^3,19,20^ of f-FTLD that include presymptomatic and symptomatic pathogenic variant carriers allows direct study of the natural history of disease using longitudinal observations. The present analysis, based on data from 2 of these large natural history studies,^19,21^ addresses limitations in previous work by incorporating longitudinal data across the disease course in participants carrying the 3 most common f-FTLD–associated pathogenic variants. Based on theoretical models^11^ and previous observational studies of Alzheimer disease^22,23^ and FTLD,^13,24^ our hypothesis was that pathogenic variants in all 3 genes would produce a nonlinear pattern of neurodegeneration, with acceleration of volume loss as patients develop symptoms.^25^ We investigated this question using longitudinal voxelwise analyses of gray matter volume and assessed whether comparable results were observed for a clinical measure of daily functioning, the Clinical Dementia Rating (CDR) plus behavioral and language domains from the National Alzheimer’s Coordinating Center (NACC) FTLD module (CDR + NACC FTLD).

## Methods

### Participants

In this longitudinal case-control study, we included 160 members of families affected by f-FTLD, most of whom were enrolled in the Advancing Research and Treatment for Frontotemporal Lobar Degeneration (ARTFL) or Longitudinal Evaluation of Familial Frontotemporal Dementia (LEFFTDS) studies, which were conducted through a consortium of 18 academic medical centers across the United States and Canada between May 2015 and September 2018. For LEFFTDS,^19^ at least 1 family member must have a pathogenic variant in the *MAPT*, *GRN*, or *C9orf72* genes. For ARTFL,^21^ families with any f-FTLD pathogenic variant or without a known pathogenic variant can enroll, but only carriers of *MAPT*, *GRN*, or *C9orf72* pathogenic variants were included in this analysis. The ARTFL and LEFFTDS protocols include annual follow-up with clinical reassessment. Additional f-FTLD cases included those enrolled in another study^26^ of FTLD at the University of California, San Francisco, and who had undergone a similar brain imaging protocol (grants AG032306 and AG019724 from the National Institutes of Health) from January 2009 to October 2016. Exclusion and inclusion criteria are provided in the eMethods in the Supplement. Local ethics committees at each of the sites approved the study, and participants provided written informed consent. This study followed the Strengthening the Reporting of Observational Studies in Epidemiology (STROBE) reporting guideline.

The sample included 100 participants with f-FTLD with a known pathogenic variant (*MAPT^+^* [28 individuals with *MAPT* pathogenic variants], *GRN^+^* [33 individuals with *GRN* pathogenic variants], and *C9orf72^+^* [39 individuals with *C9orf72* repeat expansions]) and 60 family members of known pathogenic variant carriers who did not carry the pathogenic variant (demographic characteristics are listed in the Table and eTable 1 in the Supplement). Participants with f-FTLD were grouped into the following 3 disease stages using CDR + NACC FTLD^27^: presymptomatic (CDR + NACC FTLD = 0 [n = 57]), mild or questionable (CDR + NACC FTLD = 0.5 [n = 15]), or symptomatic (CDR + NACC FTLD = ≥1 [n = 28]). Included were participants who had at least 2 structural magnetic resonance images within 1 of these stages (Table and eTable 1 in the Supplement); all available scans within that disease stage for each participant were used for the study. Each participant was only included in a single disease stage.

**Table.** Sample Characteristics

Characteristic	Control cases	All pathogenic variant carriers	*MAPT*	*GRN*	*C9orf72*	Group comparison^a^	*P* value	Post hoc^b^
No. of individuals (No. of visits)	60 (138)	100 (250)	28 (68)	33 (81)	39 (101)	NA	NA	NA
Age, mean (SD), y	47.51 (12.43)	50.48 (13.78)	43.97 (11.49)	56.89 (13.52)	50.50 (12.94)	*F*_2,97_ = 7.21	.001	*MAPT* < *GRN* and *C9orf72*
Educational level, mean (SD), y	15.61 (2.61)	15.35 (2.48)	15.56 (2.17)	15.40 (2.55)	15.17 (2.63)	*F*_2,97_ = 0.69	.50	NA
Sex, No./total No. (%)								
Female	36/60 (60)	53/100 (53)	23/39 (59)	19/33 (58)	11/28 (39)	NA	NA	NA
Male	24/60 (40)	47/100 (47)	16/39 (41)	14/33 (42)	17/28 (61)	χ^2^ = 4.24	.12	NA
Race/ethnicity, No./total No. (%)								
White	57/60 (95)	93/100 (93)	38/39 (97)	28/33 (85)	27/28 (96)	NA	NA	NA
Other^c^	3/60 (5)	7/100 (7)	1/39 (3)	5/33 (15)	1/28 (4)	NA	NA	NA
Functional severity, No. of individuals (No. of visits)								
CDR + NACC FTLD = 0	60 (138)	57 (142)	19 (47)	18 (42)	20 (53)	NA	NA	NA
CDR + NACC FTLD = 0.5	NA	15 (35)	4 (9)	6 (14)	5 (12)	NA	NA	NA
CDR + NACC FTLD = ≥1^d^	NA	28 (73)	5 (12)	9 (25)	14 (36)	NA	NA	NA
CDR + NACC FTLD = 1	NA	12 (31)	2 (5)	7 (18)	3 (8)	NA	NA	NA
CDR + NACC FTLD = 2	NA	14 (37)	2 (4)	2 (7)	10 (26)	NA	NA	NA
CDR + NACC FTLD = 3	NA	2 (5)	1 (3)	0	1 (2)	NA	NA	NA

Abbreviations: CDR + NACC FTLD, Clinical Dementia Rating plus National Alzheimer’s Coordinating Center Frontotemporal Lobar Degeneration; NA, not applicable.

^a^ *MAPT*, *GRN*, and *C9orf72* groups were compared on age and educational level using regression and on sex using χ^2^ test.

^b^ Post hoc comparisons reported if *P* < .05 for group difference.

^c^ Other includes Native American, Asian, Asian Indian, Mixed, and not reported. These groups were combined to protect confidentiality.

^d^ The 3 rows below are the detailed breakdown for CDR + NACC FTLD = ≥1.

### Clinical Assessment

The multidisciplinary assessment included neurological history and examination and collateral interview. Neuropsychological tests included the Uniform Data Set (version 3.0) neuropsychological battery.^28^ Functional status was quantified using CDR + NACC FTLD^27,29,30^ (details are provided in the Supplement). Brain imaging was not used for diagnosis or severity rating. Clinical diagnoses are listed in eTable 2 in the Supplement. All participants had genetic testing at the University of California, Los Angeles, using published methods^31^ (specific pathogenic variants are provided in the eAppendix in the Supplement).

### Neuroimaging

#### Image Acquisition

Participants underwent 3 tesla (3-T) imaging on MRI scanners (scanner types are listed in eTable 3 in the Supplement). A standard imaging protocol was used across all centers and was managed and reviewed for quality by a core group (including K.K.) at the Mayo Clinic, Rochester, Minnesota. Details of image acquisition, processing, and harmonization are provided in the eMethods in the Supplement and have been published elsewhere.^8^ All participants except 3 were scanned on the same scanner at all visits (for 2 participants, the scanner was upgraded; the third changed sites).

#### Bayesian Voxelwise Mixed-Effects Modeling

Group-level and participant-level rates of atrophy at each brain voxel were longitudinally modeled as a function of age using a bayesian hierarchical mixed-effects framework^32^ introduced by Friston and colleagues^33^ and reproduced in our in-house software suite at the Memory and Aging Center, University of California, San Francisco. The model consists of the following 2 hierarchical levels: (1) a single-participant level for individual structural trajectories and (2) a group level for an ensemble of trajectories (eMethods in the Supplement). Researchers interested in the code for the bayesian mixed-effects models can find information in the publication by Zeigler and colleagues^32^ or may contact the corresponding author of this study.

### Statistical Analysis

Details of the analytic approach are provided in the eMethods in the Supplement. To address the main hypothesis that f-FTLD pathogenic variants are associated with high rates of volume loss that increase with disease stage, we examined voxelwise maps of rates of annualized brain volume loss at each disease stage in each genetic group and compared these with rates in the control group. We fit a 3-way interaction model at each voxel as the rate of atrophy by disease stage by gene. Statistically significant voxels indicated that the association of increasing disease stage with volume loss is moderated by gene. Voxelwise maps showing where rates of volume loss were statistically significantly increased in the pathogenic variant carrier groups compared with the control group were produced using the FMRIB Software Library.^34,35^ To understand the cumulative associations of volume loss, we analyzed cross-sectional volume using the last observation for all participants in their disease stage.^34^ *P* < .05 was considered statistically significant, and all tests were 2 tailed.

To summarize rates of volume loss in various brain regions, we analyzed data for several large regions of interest (ROIs),^36^ including bilateral frontal, temporal, parietal, and occipital lobes and the thalamus and cerebellum. Thalamic and cerebellar ROIs were chosen because of their involvement in f-FTLD.^3,6^ For each ROI, we extracted the specific slope of each participant.

To examine patterns of change in clinical measures, we created linear mixed-effects regression models using participant-specific rates of change in CDR + NACC FTLD box score as the dependent variable. Higher box scores indicate more severe functional impairment. Analysis of clinical data was performed using Stata, version 14.2 (StataCorp LLC).

## Results

The sample included 100 participants with f-FTLD; the mean (SD) age was 50.48 (13.78) years, 53 (53%) were female, and 47 (47%) were male. Noncarriers made up a control group with otherwise similar genetic and environmental backgrounds compared with the carriers. The control group included 60 family members; the mean (SD) age was 47.51 (12.43) years, 36 (60%) were female, and 24 (40%) were male.

### Longitudinal Atrophy Rates

**Figure 1.**
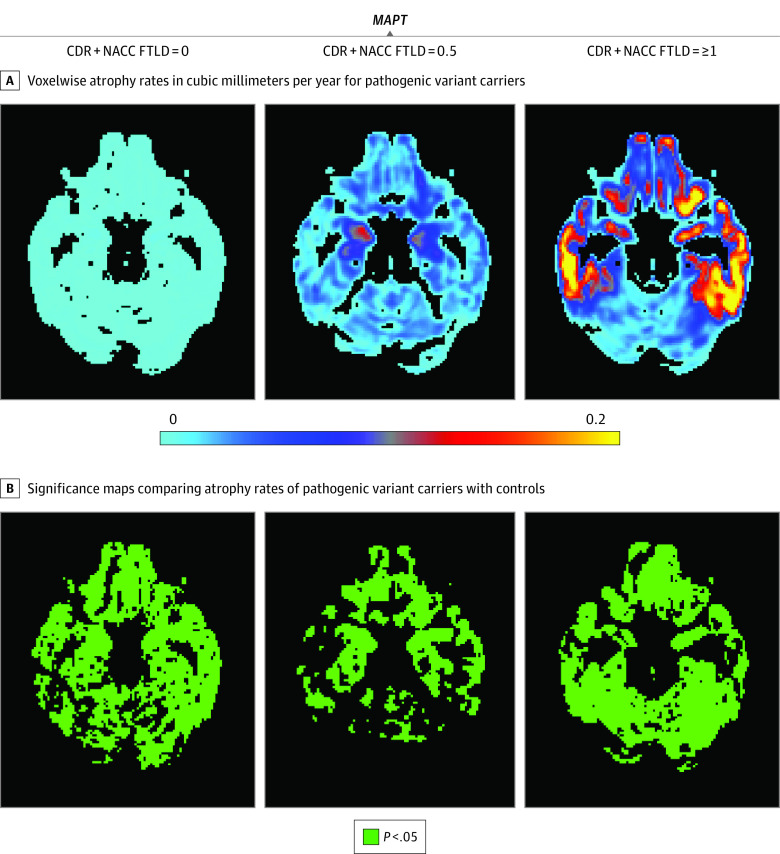
Maps of Voxelwise Atrophy Rate in *MAPT* Pathogenic Variant Carriers at 3 Levels of Disease Severity A, More positive values represent faster rates of atrophy. Based on our hypothesis, only those voxels that show rates of atrophy are presented; extending the color scale to voxels that were estimated to show volume growth would decrease interpretability by compressing the color scale in voxels of interest (those showing volume loss). B, Green voxels are statistically significant at *P* < .05 after familywise error correction for multiple comparison at each voxel. Statistically significant increased rates of volume loss compared with controls were observed at all stages. Statistically significant regions of accelerated volume loss were identified in temporal regions bilaterally in the presymptomatic stage and mild or questionable stage, with global spread in the symptomatic stage; the largest effect sizes were observed in the frontal and temporal lobes. CDR + NACC FTLD indicates Clinical Dementia Rating plus National Alzheimer’s Coordinating Center Frontotemporal Lobar Degeneration.

**Figure 2.**
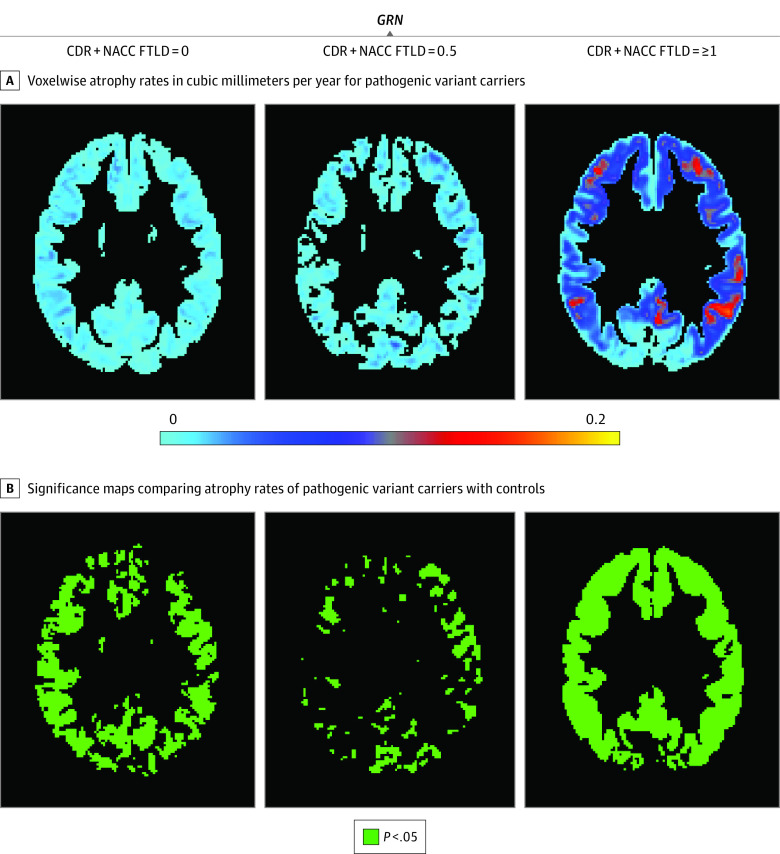
Maps of Voxelwise Atrophy Rate in *GRN* Pathogenic Variant Carriers at 3 Levels of Disease Severity A, More positive values represent faster rates of atrophy. Based on our hypothesis, only those voxels that show rates of atrophy are presented; extending the color scale to voxels that were estimated to show volume growth would decrease interpretability by compressing the color scale in voxels of interest (those showing volume loss). B, Green voxels are statistically significant at *P* < .05 after familywise error correction for multiple comparison at each voxel. Statistically significant increased rates of volume loss compared with controls were observed at all stages. In *GRN*^+^, the rate of volume loss was fairly uniform across the brain, with little evidence of acceleration between the presymptomatic stage and mild or questionable stage except for a possible area of accelerated atrophy in the putamen. With development of dementia, *GRN*^+^ showed accelerated loss of volume in portions of the frontal, temporal, and parietal lobes bilaterally. CDR + NACC FTLD indicates Clinical Dementia Rating plus National Alzheimer’s Coordinating Center Frontotemporal Lobar Degeneration.

**Figure 3.**
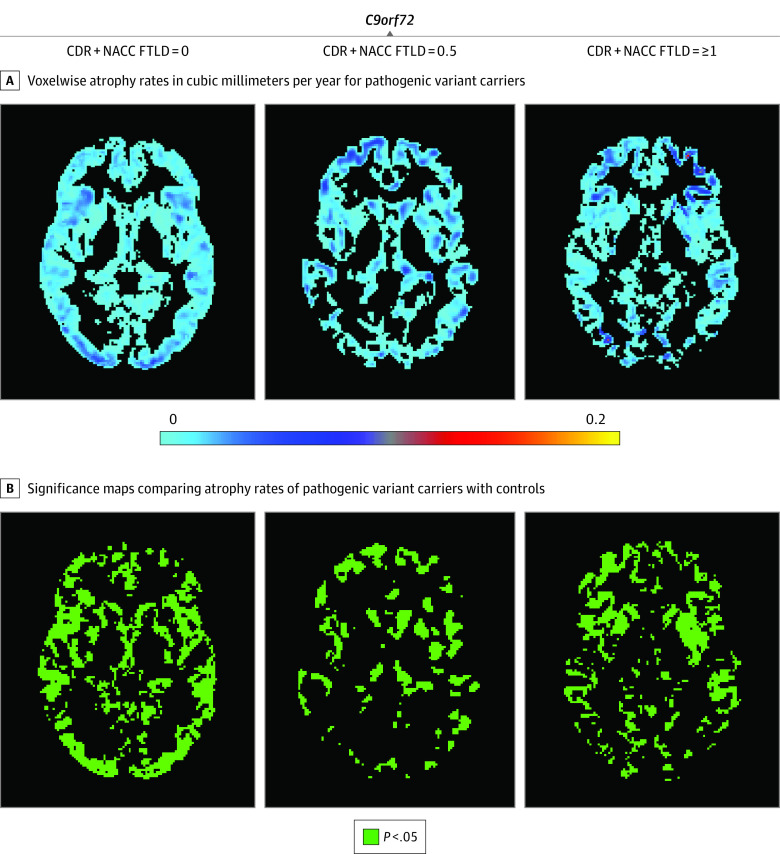
Maps of Voxelwise Atrophy Rate in *C9orf72* Repeat Expansion Carriers at 3 Levels of Disease Severity A, More positive values represent faster rates of atrophy. Based on our hypothesis, only those voxels that show rates of atrophy are presented; extending the color scale to voxels that were estimated to show volume growth would decrease interpretability by compressing the color scale in voxels of interest (those showing volume loss). B, Green voxels are statistically significant at *P* < .05 after familywise error correction for multiple comparison at each voxel. Statistically significant increased rates of volume loss compared with controls were observed at all stages. In contrast to carriers of pathogenic variants in the other 2 genes, *C9orf72* repeat expansion carriers showed little acceleration across disease stages, even with transition to dementia. Regions with the largest effect sizes were distributed among frontal, temporal, and parietal regions in *C9orf72*^+^*.* CDR + NACC FTLD indicates Clinical Dementia Rating plus National Alzheimer’s Coordinating Center Frontotemporal Lobar Degeneration.

Maps of annualized rates of atrophy (Figures 1, 2, and 3 and eFigure 1 in the Supplement) revealed statistically significant increases in the rate of volume loss for pathogenic variant carriers compared with control cases for all genes at all stages. The mean (SD) regional rates of atrophy for control participants (eTable 4 in the Supplement) were as follows for 6 lobes of interest: −170 (12) mm^3^ per year for left frontal, −160 (15) mm^3^ per year for right frontal, −77 (13) mm^3^ per year for left frontal, −73 (17) mm^3^ per year for right temporal, −105 (14) mm^3^ per year for left parietal, and −102 (16) mm^3^ per year for right parietal.

In *MAPT*^+^ carriers, statistically significant regions of accelerated volume loss compared with controls (*P* < .05 for all) were identified in temporal regions bilaterally in the presymptomatic stage. In the ROI analysis, mean (SD) values were −231 (47) mm^3^ per year for left temporal and −150 (36) mm^3^ per year for right temporal lobe. For the mild or questionable stage, the mean (SD) values were −381 (208) mm^3^ per year for left temporal and −315 (201) mm^3^ per year for right temporal lobe.There was global spread in the symptomatic stage. The largest effect sizes were observed in the frontal and temporal lobes (Figure 1). The mean (SD) values were −2269 (1574) mm^3^ per year for the left frontal lobe, −2053 (2006) mm^3^ per year for right frontal lobe, −1485 (1025) mm^3^ per year for left temporal, and −1164 (882) mm^3^ per year for right temporal lobe.

In *GRN*^+^ carriers, the rate of volume loss was fairly uniform across the brain in the presymptomatic and mild stages, with little evidence of acceleration between stages. For example, in the ROI analysis, annualized right frontal volume loss was −267 (81) mm^3^ per year in the presymptomatic stage and −182 (90) mm^3^ per year in the mild stage. The exception was a possible area of accelerated atrophy in the putamen (Figure 2). With development of dementia, *GRN*^+^ carriers showed accelerated loss of volume in portions of the frontal (mean [SD], −1530 [388] mm^3^ per year for left frontal and −1169 [555] mm^3^ per year for right frontal ROIs), temporal (mean [SD], −867 [308] mm^3^ per year for left temporal and −433 [119] mm^3^ per year for right temporal ROIs), and parietal (mean [SD], −896 [217] mm^3^ per year for left parietal and −484 [108] mm^3^ per year for right parietal ROIs) lobes bilaterally.

In contrast to pathogenic variants in the other 2 genes, *C9orf72*^+^ carriers showed minimal increase in atrophy rates across disease stages (Figure 3). For example, in the ROI analysis, the mean (SD) annualized right frontal lobe volume loss was −272 (118) mm^3^ per year in the presymptomatic stage, −310 (189) mm^3^ per year in the mild or questionable stage, and −251 (145) mm^3^ per year in the symptomatic stage. Regions with the largest effect sizes were distributed among frontal (mean [SD], −285 [199] mm^3^ per year for left frontal and −251 [145] mm^3^ per year for right frontal ROIs), temporal (mean [SD], −77 [44] mm^3^ per year for left temporal and −64 [46] mm^3^ per year for right temporal ROIs), and parietal (mean [SD], −122 [157] mm^3^ per year for left parietal and −124 [160] mm^3^ per year for right parietal ROIs) regions in *C9orf72*^+^*.*

Because the maps of volume loss indicated differences in rates of stage-dependent volume loss across groups, we fit an omnibus, disease stage by gene interaction model for rates of volume loss at each voxel. Almost every voxel in the brain (91% [247 910 of 273 039 voxels]) showed a statistically significant interaction (eFigure 2 in the Supplement), indicating that the association of disease severity with atrophy rates differs across genetic groups.

**Figure 4.**
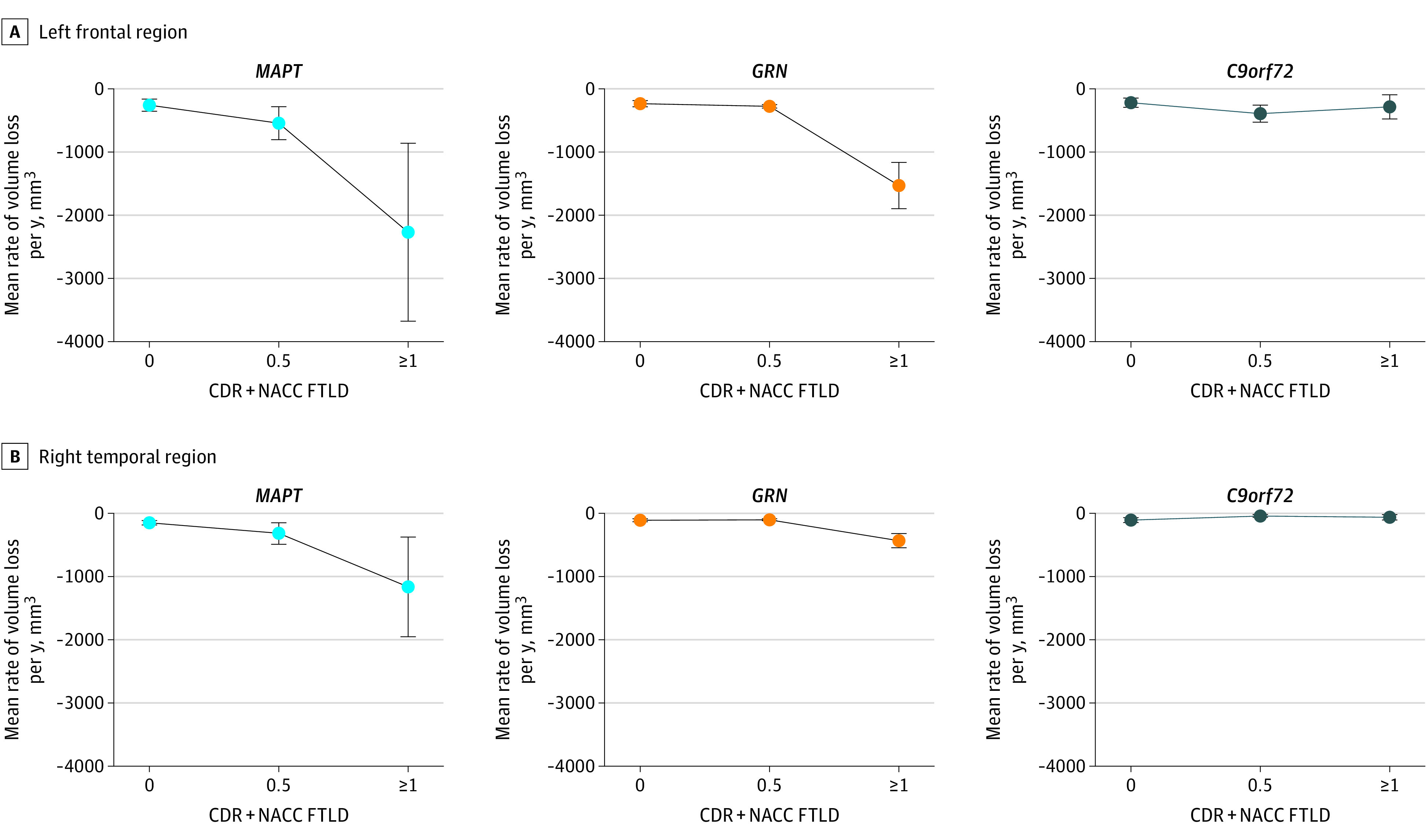
Mean Rates of Volume Loss for Frontal and Temporal Regions of Interest Examination of mean rates of volume loss in several regions of interest highlights how the consequences of disease stage vary by genetic group, with *C9orf72* repeat expansion carriers showing the least increase in the rate of atrophy as disease severity increases. *GRN* pathogenic variant carriers showed almost no differences in the rate of volume loss between CDR + NACC FTLD 0 and 0.5 stages, whereas a large increase in the rate of volume loss was observed between the 0.5 and 1 or greater stages. CDR + NACC FTLD indicates Clinical Dementia Rating plus National Alzheimer’s Coordinating Center Frontotemporal Lobar Degeneration. Error bars indicate SDs.

The ROI analysis highlighted the increases in the rate of volume loss for *MAPT*^+^ between the presymptomatic and mild or questionable stages in the right (mean [SD], −277 [119] mm^3^ per year for presymptomatic and −576 [276] for mild or questionable) and left (mean [SD], −259 [99] mm^3^ per year for presymptomatic and −544 [301] mm^3^ per year for mild or questionable) frontal, temporal (eg, mean [SD], −231 [47] mm^3^ per year for presymptomatic and −381 [208] for mild or questionable for left temporal), and parietal (eg, mean [SD], −139 [27] mm^3^ per year for presymptomatic and −303 [151] for mild or questionable for left parietal) regions (Figure 4 and eFigure 3 and eTable 4 in the Supplement). Smaller increases in the rate of volume loss with increasing disease severity were observed in the occipital lobes (mean [SD], −38 [10] for presymptomatic and −110 [68] for mild or questionable) and thalamus (mean [SD], −13 [13] for presymptomatic and −78 [50] for mild or questionable). The ROI analysis also underscored how the genetic groups differed in the degree of increased atrophy when transitioning from the mild to symptomatic stage. Even in regions where the rate of volume loss increased between the mild and symptomatic stages in *C9orf72*^+^ carriers, the magnitude of acceleration of atrophy between these 2 stages was much higher in *MAPT*^+^ and *GRN*^+^ carriers. For example, in the right frontal lobe, the increase in atrophy rate between the 2 stages was about 6 to 9 times higher in *GRN*^+^ (mean [SD], −182 [90] for mild or questionable and −1169 [555] for symptomatic) and *MAPT*^+^ (mean [SD], −576 [276] for mild or questionable and −2053 [2006] for symptomatic) carriers, respectively, compared with *C9orf72*^+^ (mean [SD], −310 [189] for mild or questionable and −251 [145] for symptomatic) carriers (eTable 4 in the Supplement). Overall, these data supported the voxelwise pattern of results.

One potential reason why the rate of volume loss may appear erroneously low in the *C9orf72*^+^ group is that the spatial location of atrophy may vary across *C9orf72*^+^ carriers, such that mean rates of change in any single region might be low at the group level. We examined this question by creating voxelwise maps of variance in rates of change (eFigure 4 in the Supplement) and by plotting mean lobar rates of change for each pathogenic variant carrier (eFigure 5 in the Supplement) for each genetic group at each stage. We also created maps of annualized volume loss for each individual in the CDR + NACC FTLD = 1 or greater stage (eFigure 6 in the Supplement). These maps and plots revealed that variability was highest in *MAPT*^+^ and lowest in *C9orf72*^+^ carriers, suggesting that excessive variability across *C9orf72*^+^ carriers, either in the spatial location of atrophy or the rate of atrophy, does not account for the group-level findings.

### Clinical Decline

Rates of functional decline, as measured by the CDR + NACC FTLD box score, showed a disease stage by gene interaction, similar to the rates of atrophy (bottom of eTable 4 in the Supplement). In contrast to the imaging results, the *C9orf72*^+^ and *GRN*^+^ groups showed similar differences in the rate of change from 0 (mean [SD], 0.1 [0] box score units per year for *C9orf72*^+^ and 0.1 [0] box score units per year for *GRN*^+^) to 0.5 (mean [SD], 0.4 [0.1] box score units per year for *C9orf72*^+^ and 0.3 [0.2] box score units per year for *GRN*^+^) and from 0.5 to 1 (mean [SD], 1.5 [0.3] box score units per year for *C9orf72*^+^ and 1.4 [0.5] box score units per year for *GRN*^+^). The estimated difference in rate of clinical decline from 0.5 (mean [SD], 0.3 [0.1] for *MAPT*^+^) to 1 (mean [SD], 2.2 [1.0] for *MAPT*^+^) was almost twice as large in the *MAPT*^+^ group as it was for *GRN*^+^ or *C9orf72*^+^ groups, consistent with neuroimaging.

### Cross-sectional Atrophy

The small increases in the rate of volume loss in the *C9orf72*^+^ group prompted the question of whether expansions in this gene are associated with accumulation of brain atrophy to a similar degree as pathogenic variants in the other genes. Voxelwise maps depicting cross-sectional atrophy at each stage in each gene are shown in eFigure 7A, C, and E in the Supplement, with maps of statistical significance shown in eFigure 7B, D, and F in the Supplement. At CDR + NACC FTLD = 1 or greater, all groups showed more atrophy in all ROIs compared with the control group (eFigure 8 and eTable 5 in the Supplement). The *MAPT*^+^ group showed the greatest degree of frontal (mean [SD] volume, left frontal: 12 683 [2345] mm^3^; right frontal: 13 235 [2015] mm^3^) and temporal (mean [SD] volume, left temporal: 8652 [1090] mm^3^; right temporal: 8628 [1237] mm^3^) atrophy at this stage, followed by *GRN*^+^ (eg, mean [SD] volume, right frontal: 13 679 [2448] mm^3^; right temporal: 9271 [1530] mm^3^) and *C9orf72*^+^ groups (eg, mean [SD] volume, right frontal: 14 012 [1485] mm^3^; right temporal: 9336 [734] mm^3^), with similar degrees of atrophy in *GRN*^+^ and *C9orf72*^+^ groups.

## Discussion

The objective of this study was to characterize the evolution of neurodegeneration in FTLD associated with pathogenic variants in 3 different genes. Consistent with our hypothesis, we found evidence for acceleration of neurodegeneration, as measured by loss of brain volume, in *MAPT*^+^ and *GRN*^+^. Compared with these 2 genetic groups, *C9orf72*^+^ was associated with attenuated increases in the rate of volume loss, even with transition to dementia. The differences in mean rates of change across groups were not accounted for by differences in interparticipant variability. Despite differences in patterns of acceleration, cross-sectional maps of atrophy indicated that pathogenic variants in all 3 genes were associated with substantial accumulation of atrophy by the time patients developed dementia. Progression in a clinical measure of disease severity diverged from this pattern, with the rate of functional decline in *C9orf72*^+^ being similar to that in *GRN*^+^. Together, these findings suggest that, although the destiny for the brain in *C9orf72*^+^ is similar to that of other pathogenic variants, the path to this point is different, being slower and more constant over time. This finding has implications for models that would target prediction of symptom onset or tracking of disease progression. In addition, it raises important questions about the unique pathophysiology associated with *C9orf72* repeat expansions and how this finding relates to symptoms.

These results have several implications for work predicated on accurate prognostication. Treatment studies enrolling groups with f-FTLD must consider the heterogeneity conferred by both the disease stage and the altered gene. In addition, a critical goal for trials in f-FTLD is to identify predictors for when symptoms will develop so that participants who are close to symptom onset and demonstrate delay in this transition can be enrolled.^37,38,39^ Recent publications from studies of f-FTLD and other familial neurodegenerative diseases indicate that cross-sectional^40^ and longitudinal^5^ measurements of imaging and fluid biomarkers can predict development of symptoms. Our results suggest that models assuming rapid change in biomarkers preceding or accompanying development of symptoms may apply well to *MAPT*^+^ and *GRN*^+^ carriers, but not as well to *C9orf72*^+^ carriers. However, whereas the nonlinear nature of change in *MAPT*^+^ and *GRN*^+^ may make it difficult to predict onset of symptoms using measures collected in the stable or asymptomatic phase, such measurements may be more useful in *C9orf72*^+^, where decline is more linear.

Different dynamics of change across these 3 genetic groups may be associated with the unique pathophysiology of pathogenic variants in each gene. We observed regions of accelerated volume loss in the medial temporal regions relative to the rest of the brain in *MAPT*^+^ early in the course of illness. This finding indicates fairly consistent associations in this region across participants, consistent with prior literature,^3,4,13^ indicating that the medial temporal lobes are particularly vulnerable to *MAPT* pathogenic variants. The *MAPT* pathogenic variants lead to accumulation of modified tau molecules that damage neurons, although the mechanisms are not completely understood,^41^ and our model suggests that the associations of interventions in the early stages of disease might be measurable in reduced rates of volume loss in medial temporal regions or reduced spread of atrophy to other regions. Compared with the other genetic groups, *MAPT*^+^ tends to exhibit atrophy more focally and symmetrically, which could improve the power to detect atrophy at the group level.

In contrast to *MAPT*^+^, little acceleration of volume loss occurred in any region until symptom onset in *GRN*^+^. This observation is consistent with studies showing minimal cross-sectional^13,42^ or longitudinal^2,15,42^ atrophy in presymptomatic *GRN*^+^. Moreover, other studies have shown that rapid neuroimaging changes^13^ and 3-fold to 4-fold increases in cerebrospinal fluid neurofilament light chain levels^43^ occur around the time of symptom onset in *GRN*^+^. The primary consequence of the *GRN* pathogenic variant is reduced production of the progranulin protein. This reduction is detectable early in life, and levels of progranulin are similar in the presymptomatic and symptomatic stages, indicating that progranulin reduction may not be directly responsible for symptoms.^44,45,46^ These observations could be consistent with the theory that a secondary biological process (“hit”) occurring in the context of low progranulin sets off a rapid cascade of neurodegeneration^13^ or that there is a tipping point in the accumulation of cellular or tissue damage. If this 2-hit model does indeed apply to *GRN*^+^, progranulin-raising medications administered in the presymptomatic stage may delay onset of symptoms but might have only a minimal impact on measurable imaging changes in this phase.

The observation that *C9orf72*^+^ showed only a small degree of acceleration yet the degree of volume loss accumulated was close to the amount seen with *GRN*^+^ in the symptomatic phase might suggest that atrophy starts at a younger age, which is supported by previous studies.^6,12^ Furthermore, studies^15,47,48,49^ of small cohorts of *C9orf72*^+^ have highlighted slow progression with insidious transition from presymptomatic to symptomatic phases. Our findings indicate that this insidious transition may be a common feature of disease associated with *C9orf72* repeat expansions, although rapid deterioration may still occur in some cases or later in the illness.^50^ Divergence in rates of volume loss and clinical decline in *C9orf72*^+^ is consistent with prior findings suggesting that neuronal dysfunction (particularly salience network and medial pulvinar dysfunction quantified with task-free functional magnetic resonance imaging) rather than global neuronal loss may best predict clinical severity in *C9orf72*^+^.^49^

### Limitations

These results should be interpreted in the context of several limitations. First, because of the rarity of this disease, the sample sizes are small. Although the longitudinal nature of this study improves our ability to directly quantify changes, replication will be important given the small sample sizes in some of the groups. A second consequence of the small sample size is that, although we separated participants into 3 genetic groups, we were unable to look at the association of specific pathogenic variants, which produce overlapping but distinct atrophy patterns^51,52^ and different disease durations.^20^ We addressed this limitation in part by producing variability maps to understand the consequences of within-group heterogeneity. Third, the small sample size required careful consideration of covariates, and we were unable to fully explore all potential factors, such as sex. This limitation is a topic that will be the focus of future investigation.

## Conclusions

To our knowledge, this investigation is the first study to analyze the natural history of longitudinal volumetric changes in pathogenic variant carriers in 3 genes, across the entire disease spectrum. This study advances the knowledge of between-gene differences in atrophy rates as a function of disease severity, and the results have implications for clinical trial design. These findings suggest that the mechanism by which *C9orf72* pathogenic variants engender symptoms may be fundamentally different from the mechanisms associated with *MAPT* and *GRN* pathogenic variants.

## Notes

Supplement. **eMethods.** Supplemental Methods **eAppendix.** Specific MAPT and GRN Mutations Included in This Study **eFigure 1.** Maps of Voxel-Wise Atrophy Rate in Each Genetic Group at Three Levels of Disease Severity **eFigure 2.** Voxel-Wise Mutation Type by Disease Severity Interaction **eFigure 3.** Mean Rates of Volume Loss for Several Regions of Interest **eFigure 4.** SD of Velocity Maps in Each Genetic Group at Three Levels of Disease Severity **eFigure 5.** Individual Variability in Mean Rates of Atrophy for Several Regions of Interest **eFigure 6.** Individual Maps of Voxel-Wise Atrophy Rates for All Symptomatic Mutation Carriers **eFigure 7.** Cross-Sectional Atrophy Maps in Each Genetic Group at Three Levels of Disease Severity **eFigure 8.** Cross-Sectional Atrophy by Region of Interest **eTable 1.** Demographic Information for Each Subgroup **eTable 2.** Diagnostic Composition **eTable 3.** Distribution of Scanner Vendors by Genetic Group **eTable 4.** Mean and SD of Annualized Rate of Volume Loss **eTable 5.** Cross-sectional Volume by Region of Interest
