# Supplementary material for: Rates of Brain Atrophy Across Disease Stages in Familial Frontotemporal Dementia Associated With MAPT, GRN, and C9orf72 Pathogenic Variants
Source: JAMA Netw Open. 2020 Oct 28;3(10):e2022847. doi: 10.1001/jamanetworkopen.2020.22847 (PMC7593814; doi:10.1001/jamanetworkopen.2020.22847)
Supplement: Supplement. — eMethods. Supplemental Methods eAppendix. Specific MAPT and GRN Mutations Included in This Study eFigure 1. Maps of Voxel-Wise Atrophy Rate in Each Genetic Group at Three Levels of Disease Severity eFigure 2. Voxel-Wise Mutation Type by Disease Severity Interaction eFigure 3. Mean Rates of Volume Loss for Several Regions of Interest eFigure 4. SD of Velocity Maps in Each Genetic Group at Three Levels of Disease Severity eFigure 5. Individual Variability in Mean Rates of Atrophy for Several Regions of Interest eFigure 6. Individual Maps of Voxel-Wise Atrophy Rates for All Symptomatic Mutation Carriers eFigure 7. Cross-Sectional Atrophy Maps in Each Genetic Group at Three Levels of Disease Severity eFigure 8. Cross-Sectional Atrophy by Region of Interest eTable 1. Demographic Information for Each Subgroup eTable 2. Diagnostic Composition eTable 3. Distribution of Scanner Vendors by Genetic Group eTable 4. Mean and SD of Annualized Rate of Volume Loss eTable 5. Cross-sectional Volume by Region of Interest [file jamanetwopen-e2022847-s001.pdf]

Supplementary Online Content

Staffaroni AM, Goh SYM, Cobigo Y, et al; for the ARTFL-LEFFTDS Longitudinal Frontotemporal Lobar Degeneration Consortium. Rates of brain atrophy across disease stages in familial frontotemporal dementia associated with *MAPT*, *GRN*, and *C9orf72* pathogenic variants. *JAMA Netw Open*. 2020;3(10):e2022847. doi:10.1001/jamanetworkopen.2020.22847

eMethods. Supplemental Methods

eAppendix. Specific MAPT and GRN Mutations Included in This Study

eFigure 1. Maps of Voxel-Wise Atrophy Rate in Each Genetic Group at Three Levels of Disease Severity

eFigure 2. Voxel-Wise Mutation Type by Disease Severity Interaction

eFigure 3. Mean Rates of Volume Loss for Several Regions of Interest

eFigure 4. SD of Velocity Maps in Each Genetic Group at Three Levels of Disease Severity

eFigure 5. Individual Variability in Mean Rates of Atrophy for Several Regions of Interest

eFigure 6. Individual Maps of Voxel-Wise Atrophy Rates for All Symptomatic Mutation Carriers

eFigure 7. Cross-Sectional Atrophy Maps in Each Genetic Group at Three Levels of Disease Severity

eFigure 8. Cross-Sectional Atrophy by Region of Interest

eTable 1. Demographic Information for Each Subgroup

eTable 2. Diagnostic Composition

eTable 3. Distribution of Scanner Vendors by Genetic Group

eTable 4. Mean and SD of Annualized Rate of Volume Loss

eTable 5. Cross-sectional Volume by Region of Interest

© 2020 Staffaroni AM et al. *JAMA Network Open*.

This supplementary material has been provided by the authors to give readers additional information about their work.

eMethods. Supplemental Methods

Additional Inclusion and Exclusion Criteria

Additional inclusion criteria include a reliable informant who personally speaks with or sees that subject at least weekly, subject is sufficiently fluent in English to complete all measures, willing and able to consent to the protocol and undergo yearly evaluations over 3 years (baseline and at years 1, 2, and 3 after baseline), willing and able to undergo neuropsychological testing (at least at baseline visit), and no contraindication to MRI imaging. Exclusion criteria include: presence of a structural brain lesion (eg, tumor, cortical infarct), presence of another neurologic disorder which could impact findings (eg, multiple sclerosis), and unwillingness to return for follow-up yearly, undergo neuropsychological testing, MR imaging, presence of amyotrophic lateral sclerosis (ALS) at baseline, and no reliable informant.

Clinical Dementia Rating Scale (CDR®) plus Behavioral and Language Domains from the National Alzheimer's Coordinating Center (NACC) FTLD module (CDR® plus NACC FTLD)

The traditional CDR® can be used to generate a total score that represents a weighted average of six functional domain scores to categorize each patient as having questionable or mild symptoms of neurodegenerative disease (CDR® = 0.5) or clear symptoms of dementia (CDR® = 1, 2, or 3). This system is biased toward memory complaints, which are not the presenting symptom in many patients with FTLD. To classify cases for this study, we used a scoring algorithm that included the traditional CDR® domains and the Behavioral and Language domains, which is described in greater detail elsewhere [1]. In addition to the global score, we calculated the sum of

© 2020 Staffaroni AM et al. *JAMA Network Open*.

boxes at each time point; the sum of boxes is an ordinal metric (range: 0 –24) that sums scores on each of the eight domains, and has been used as a clinical measure of disease severity in prior studies of dementia [2,3].

Image Acquisition

Participants were scanned at 3 Tesla on MRI scanners from one of three vendors: Philips Medical Systems, Siemens, or General Electric Medical Systems. A standard imaging protocol was used across all centers, managed and reviewed for quality by a core group at the Mayo Clinic, Rochester, MN. Further description of the harmonization and quality control process have been described elsewhere[4]. The current analysis used the T1-weighted images, which were acquired as Magnetization Prepared Rapid Gradient Echo (MP-RAGE) images using the following parameters: 240x256x256 matrix; about 170 slices; voxel size = 1.05x1.05x1.25 mm^3; flip angle, TE and TR varied by vendor.

Prior to any preprocessing of images, all T1-weighted scans were visually inspected for quality control. Images with excessive motion or other image artifacts were excluded. T1-weighted images were bias field corrected using the nonparametric nonuniform intensity normalization (N3) algorithm[5]. Image segmentation was performed with the SPM12 unified segmentation framework (Wellcome Trust Center for Neuroimaging, London, UK).

An intra-subject template was created using non-linear diffeomorphic and rigid-body registration as proposed by the symmetric diffeomorphic registration for longitudinal MRI framework[6]. A within-subject modulation was applied by multiplying the timepoints' Jacobians with the intra-

© 2020 Staffaroni AM et al. *JAMA Network Open*.

subject averaged tissues[7]. A group template was generated from the within-subject average gray- and white-matter tissues and cerebrospinal fluid using Large Deformation Diffeomorphic Metric Mapping[8]. Modulated intra-subject gray and white matter were normalized and smoothed (4mm full width half maximum Gaussian kernel) in the group template. Each step of the transformation from native space to group template space was carefully inspected for errors.

Bayesian Mixed Effects Model

In our study, the trajectory is described as a polynomial with degree *D* of the time, i.e. the age at the *j*-th observation of subject *i* has a gray matter density (response) in one voxel y_{ij} such that

$$y_{ij} = \sum_{d=0}^{D} \theta^{(1)} t_j^d + \varepsilon_{ij}^{(1)}$$

The model was fitted using a design matrix $X^{(1)}$ built with the age t_j^d, the subjects' age at their image acquisition time. $\theta^{(1)}$ and $\varepsilon_{ij}^{(1)}$ are the first level vectors of parameters and noise, respectively. Thus, the complete model is written as $y = X^{(1)}\theta^{(1)} + \varepsilon^{(1)}$ where $X^{(1)}$ and $\varepsilon^{(1)}$ are the first level design matrix and noise, respectively. The second level is modeled as $\theta^{(1)} = X^{(2)}\theta^{(2)} + \varepsilon^{(2)}$, where $X^{(2)}$, $\theta^{(2)}$, and $\varepsilon^{(2)}$ are the second level design matrix, parameters, and noise, respectively. The second level design matrix consists of the covariates of interest at baseline. For this study, we considered age and total intracranial volume (TIV) as covariates. At each level, the noise is considered drawn from a centered Gaussian distribution: $\varepsilon^{(u)} \sim N(0, C_\varepsilon^{(u)})$, where $C_\varepsilon^{(u)}$ denotes the hierarchical level *u* covariance.

Statistical Analysis

© 2020 Staffaroni AM et al. *JAMA Network Open*.

To address the main hypothesis that f-FTLD mutations are associated with abnormally high rates of volume loss that increase with disease stage, we examined voxel-wise maps of the rates of annualized brain volume loss at each disease stage for groups with mutations in each gene and compared these with rates in controls. We also fit a three-way interaction model at each voxel: rate of atrophy x stage x gene. Significant voxels show that the effect of increasing disease stage on volume loss is moderated by gene. Voxel-wise maps depicting regions where rates of volume loss were significantly increased in the mutation carrier groups compared with controls were produced after correcting for multiple comparisons using FSL's permutation inference framework[32] with threshold-free cluster enhancement.[33] To understand the cumulative effects of decline in volume in each stage, we analyzed cross-sectional volume using the last observation for each participant in their disease stage. We compared volumes for each of the genetic groups at each of the disease stages with volumes in the control group using the FSL Randomise function[32] with permutation testing without additional covariates. *P* values less than 0.05 were considered significant and all tests were two-tailed.

To summarize the rates of volume loss in various brain regions, we analyzed data for several large ROIs derived by summing ROIs from the Desikan-Killiany atlas[34]: bilateral frontal, temporal, parietal and occipital lobes, and thalamus, and the cerebellum. Thalamic and cerebellum ROIs were chosen because of the known involvement of these regions in f-FTLD.[3,6] For each ROI, we extracted each person's subject-specific slope and report these measurements for each group. We do not perform formal statistical tests because statistically significant gene-by-stage interactions were established for these estimates within the voxelwise Bayesian mixed linear effects model and the subject-specific slopes extracted from the Bayesian models are

© 2020 Staffaroni AM et al. *JAMA Network Open*.

affected by shrinkage towards the mean, resulting in estimated individual trajectories that have been shifted closer to parallel with the group trajectory (i.e., important variance has been removed from the raw trajectories).

To examime patterns of change in clinical measures, we created linear mixed effects regression models using subject-specific rates of change in CDR®+ NACC FTLD Box Score as the dependent variable. The rate of change was extracted from a random slope, random intercept linear mixed effects model in which the only predictor was time. Again, due to shrinkage of these estimated slopes towards the mean, we present the results without calculating statistical significance.

Image processing and imaging based statistical analyses were conducted using the FMRIB Software Library (FSL; fsl.fmrib.ox.ac.uk) and the Statistical Parametric Mapping Software package (SPM; www.fil.ion.ucl.ac.uk/spm), and analysis of clinical data were performed using Stata version 14.2 (Statacorp).

1. Miyagawa T, Brushaber D, Syrjanen J, et al. Utility of the global CDR® plus NACC FTLD rating and development of scoring rules: Data from the ARTFL/LEFFTDS Consortium. *Alzheimers Dement*. 2020;16(1):106-117. doi:10.1002/alz.12033
2. Staffaroni AM, Ljubenkov PA, Kornak J, et al. Longitudinal multimodal imaging and clinical endpoints for frontotemporal dementia clinical trials. *Brain*. 2019;142(2):443-459. doi:10.1093/brain/awy319
3. Miyagawa T, Brushaber D, Syrjanen J, et al. Use of the CDR® plus NACC FTLD in mild FTLD: Data from the ARTFL/LEFFTDS consortium. *Alzheimers Dement*. August 2019.

© 2020 Staffaroni AM et al. *JAMA Network Open*.

doi:10.1016/j.jalz.2019.05.013

4. Olney NT, Ong E, Goh S-YM, et al. Clinical and volumetric changes with increasing functional impairment in familial frontotemporal lobar degeneration. *Alzheimer's Dement*. November 2019. doi:10.1016/j.jalz.2019.08.196

5. Sled JG, Zijdenbos AP, Evans AC. A nonparametric method for automatic correction of intensity nonuniformity in MRI data. *IEEE Trans Med Imaging*. 1998;17(1):87-97. doi:10.1109/42.668698

6. Ashburner J, Ridgway GR. Symmetric diffeomorphic modeling of longitudinal structural MRI. *Front Neurosci*. 2012;6:197. doi:10.3389/fnins.2012.00197

7. Ziegler G, Penny WD, Ridgway GR, Ourselin S, Friston KJ, Alzheimer's Disease Neuroimaging Initiative. Estimating anatomical trajectories with Bayesian mixed-effects modeling. *Neuroimage*. 2015;121:51-68. doi:10.1016/j.neuroimage.2015.06.094

8. Ashburner J, Friston KJ. Diffeomorphic registration using geodesic shooting and Gauss-Newton optimisation. *Neuroimage*. 2011;55(3):954-967. doi:10.1016/j.neuroimage.2010.12.049

© 2020 Staffaroni AM et al. *JAMA Network Open*.

eAppendix. Specific *MAPT* and *GRN* Mutations Included in This Study

MAPT: IVS10+16C>T (g.44087784C>T, c.1920+16C>T)
MAPT: IVS9-10G>T (g.44087666G>A, c.1828-10G>T)
MAPT: N279K (c.1842T>G, p.N614K)
MAPT: P301L (c.1907C>T, p.P636L)
MAPT: R406W (c.2221C>T, p.R741W)
MAPT: S305I (c.1919G>T, p.S640I)
MAPT: S305N (c.1919G>A, p.S640N)
MAPT: V337M (c.2014G>A, p.V672M)

GRN: A1T (c.A1T, p.Met1Leu)
GRN: A9D (c.26C>A, p.A9D)
GRN: I422fs (c.1256_1263dupGAAGCGAG, p.I422Efs*72)
GRN: IVS3+2T>C (g.42426921T>C, c.264+2T>C)
GRN: IVS8 (c.836-1G>C p.IVS8-1G>C)
GRN: E421fs (c.1263_1264insGAAGCGAG,p.E421fs)
GRN: P512fs (c.1535delC, p.P512Lfs*5)
GRN: R110X (c.328C>T, p.R110*)
GRN: R198fs (c.592_593delAG, p.R198Gfs*19)
GRN: R418X (c.1252C>T, p.R418*)
GRN: R493X (c.1477C>T, p.R493*)
GRN: S226fs (c.675_676delCA, p.S226Wfs*28)
GRN: T52fs (c.154delA, p.T52Hfs*2)
GRN: W304fs (c.910_911dupTG, p.W304Cfs*58)
GRN: Y294X (c.882T>G, p.Y294*)

© 2020 Staffaroni AM et al. *JAMA Network Open*.

eFigure 1. Maps of Voxel-Wise Atrophy Rate in Each Genetic Group at Three Levels of Disease Severity

© 2020 Staffaroni AM et al. *JAMA Network Open*.

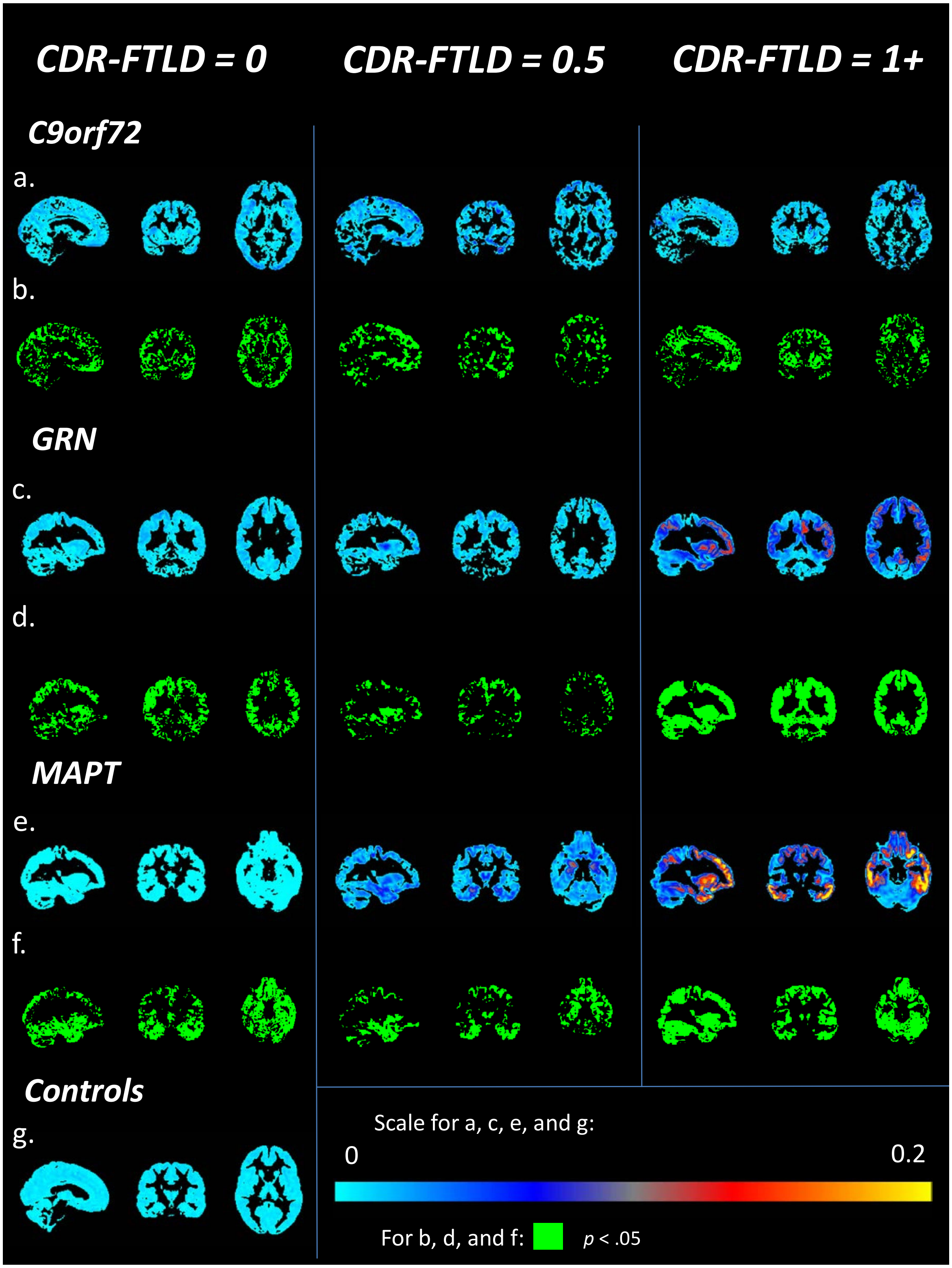

eFigure 2. Voxel-Wise Mutation Type by Disease Severity Interaction

© 2020 Staffaroni AM et al. *JAMA Network Open*.

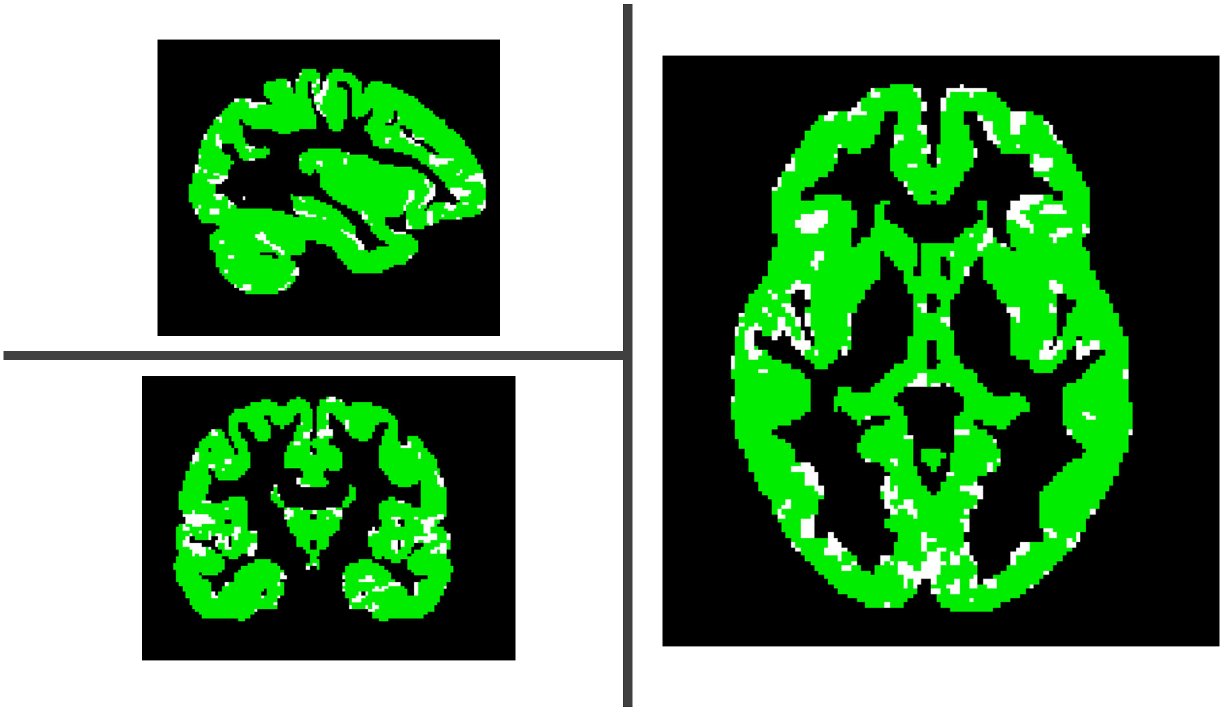

© 2020 Staffaroni AM et al. *JAMA Network Open*.

eFigure 3. Mean Rates of Volume Loss for Several Regions of Interest

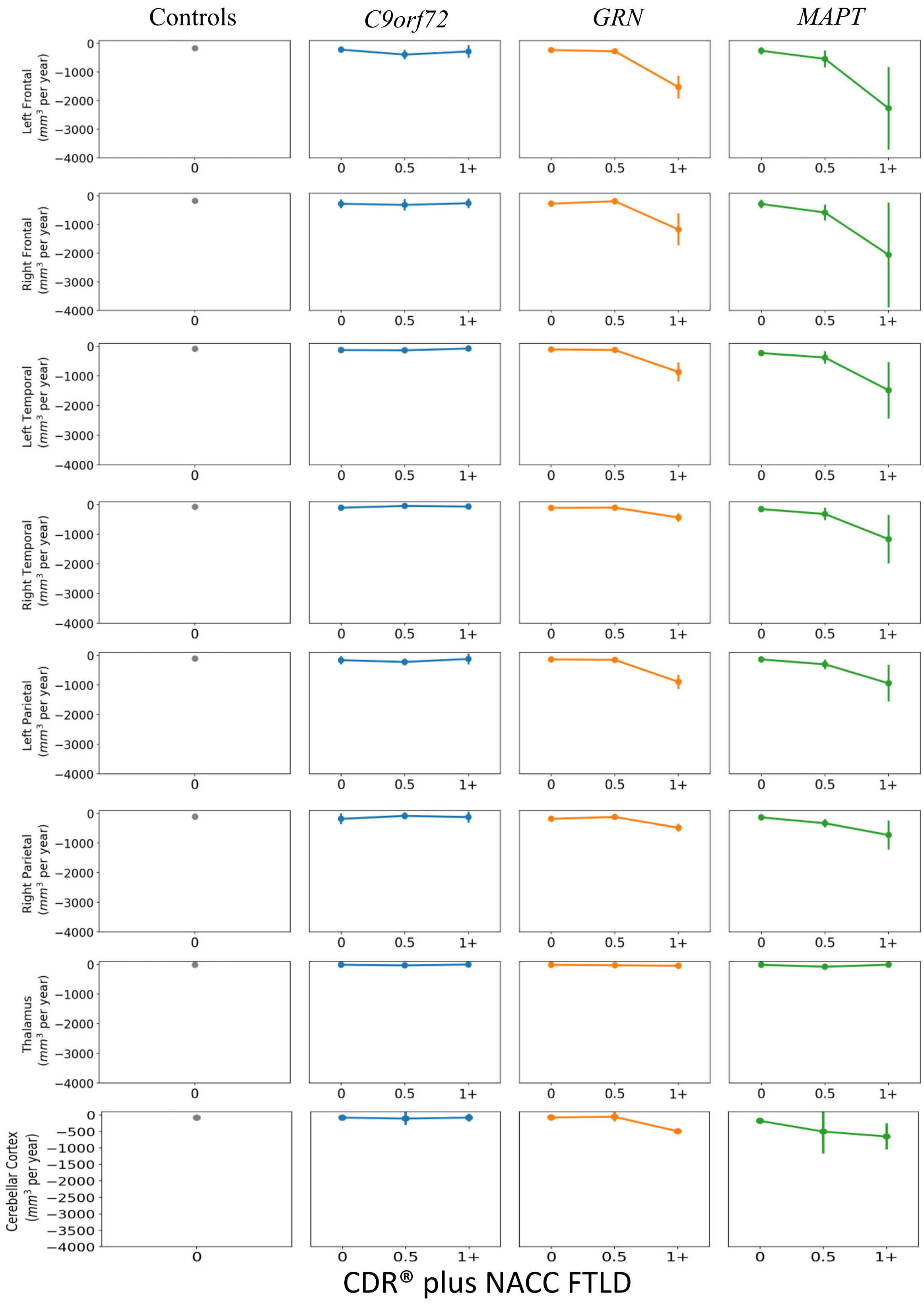

© 2020 Staffaroni AM et al. *JAMA Network Open*.

eFigure 4. SD of Velocity Maps in Each Genetic Group at Three Levels of Disease Severity

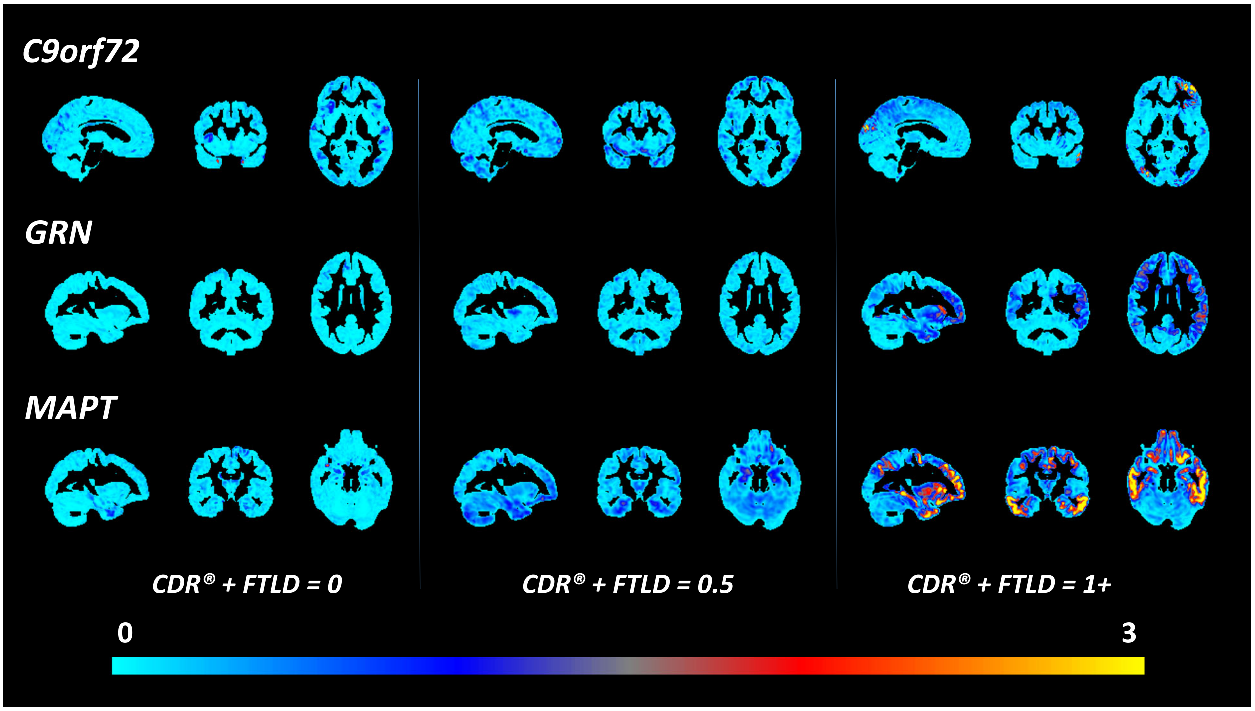

© 2020 Staffaroni AM et al. *JAMA Network Open*.

eFigure 5a. Individual Variability in Mean Rates of Atrophy for Several Regions of Interest

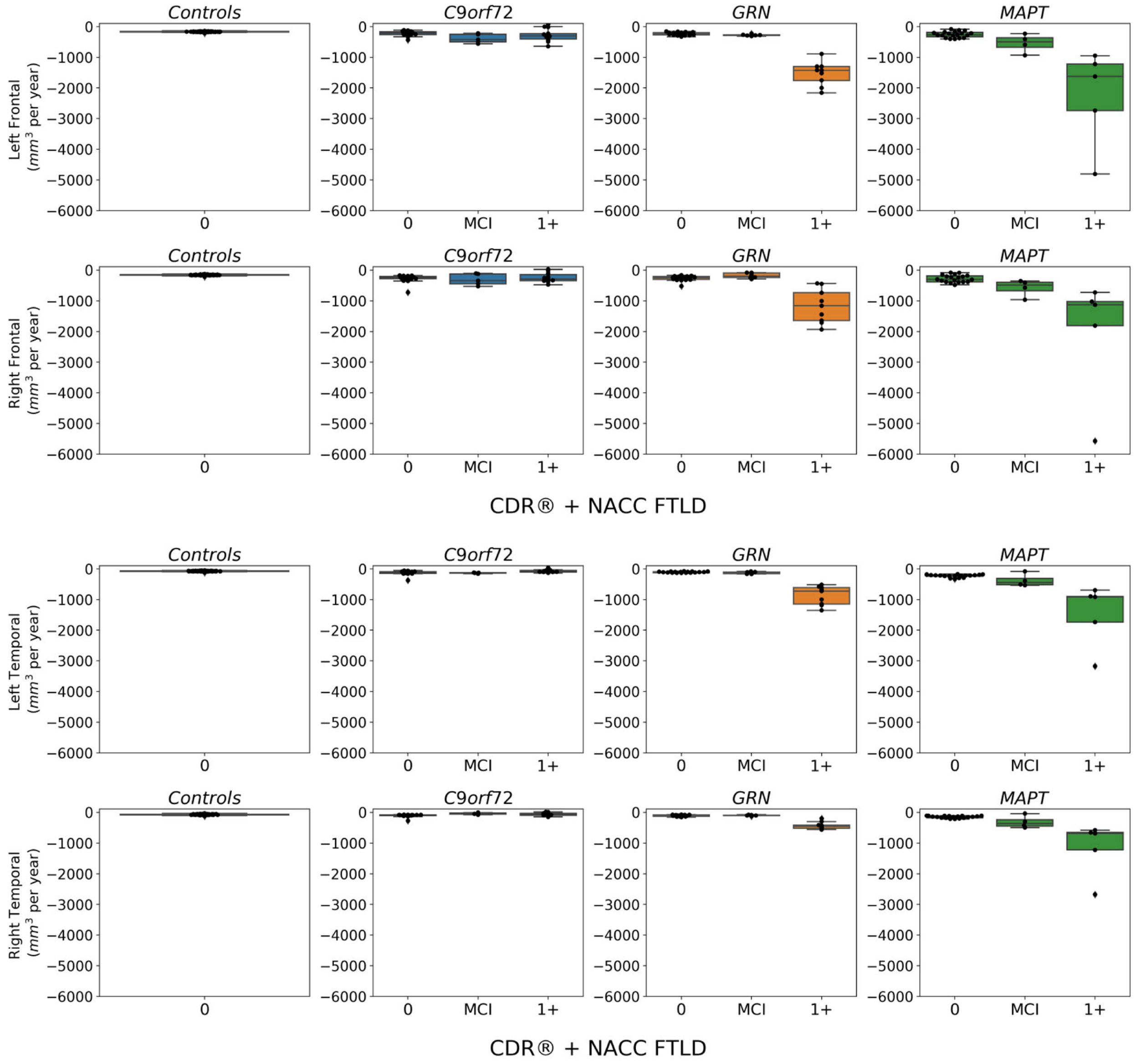

© 2020 Staffaroni AM et al. *JAMA Network Open*.

eFigure 5b. Individual Variability in Mean Rates of Atrophy for Several Regions of Interest

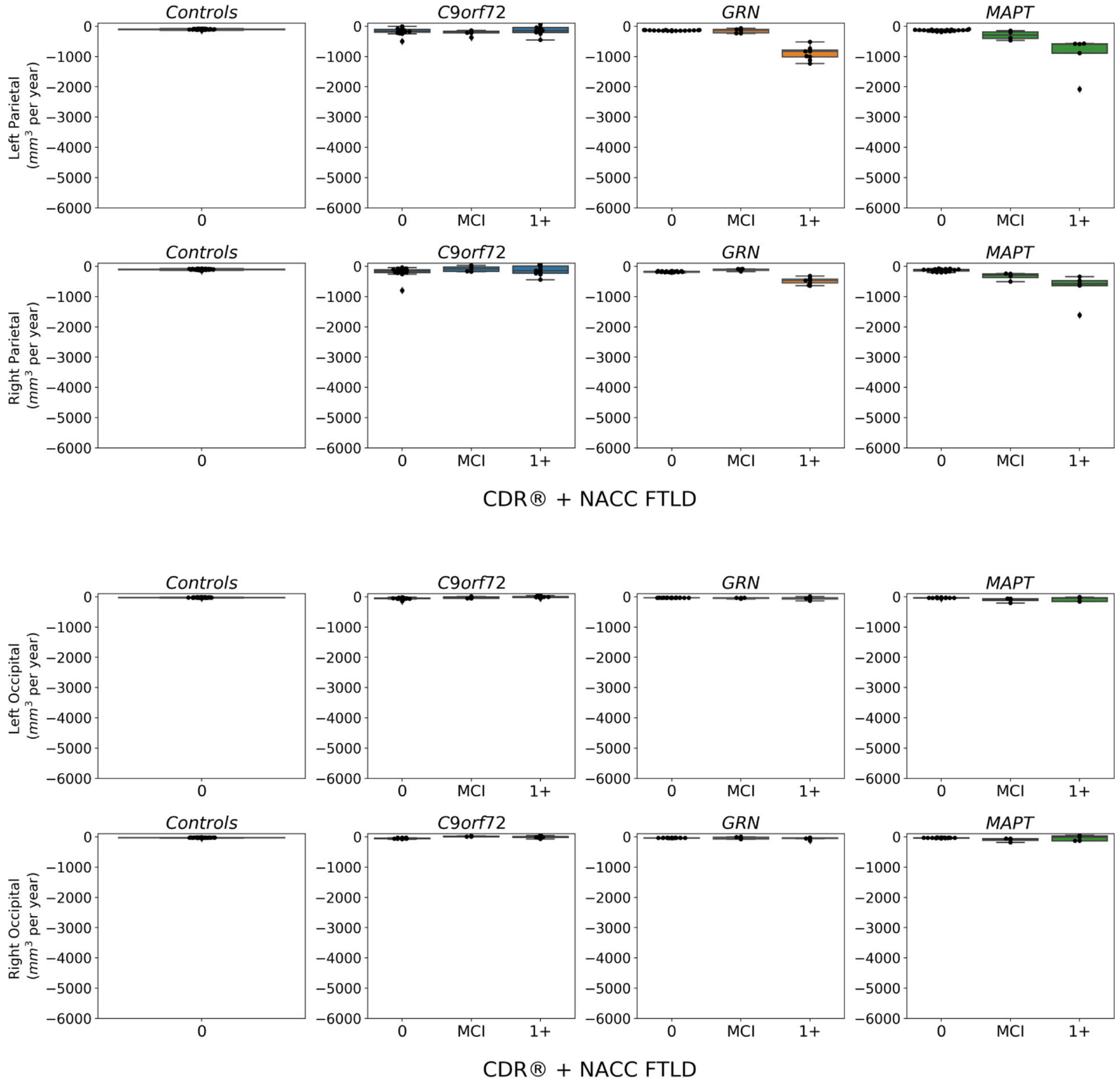

© 2020 Staffaroni AM et al. *JAMA Network Open*.

eFigure 6. Individual Maps of Voxel-Wise Atrophy Rates for All Symptomatic Mutation Carriers

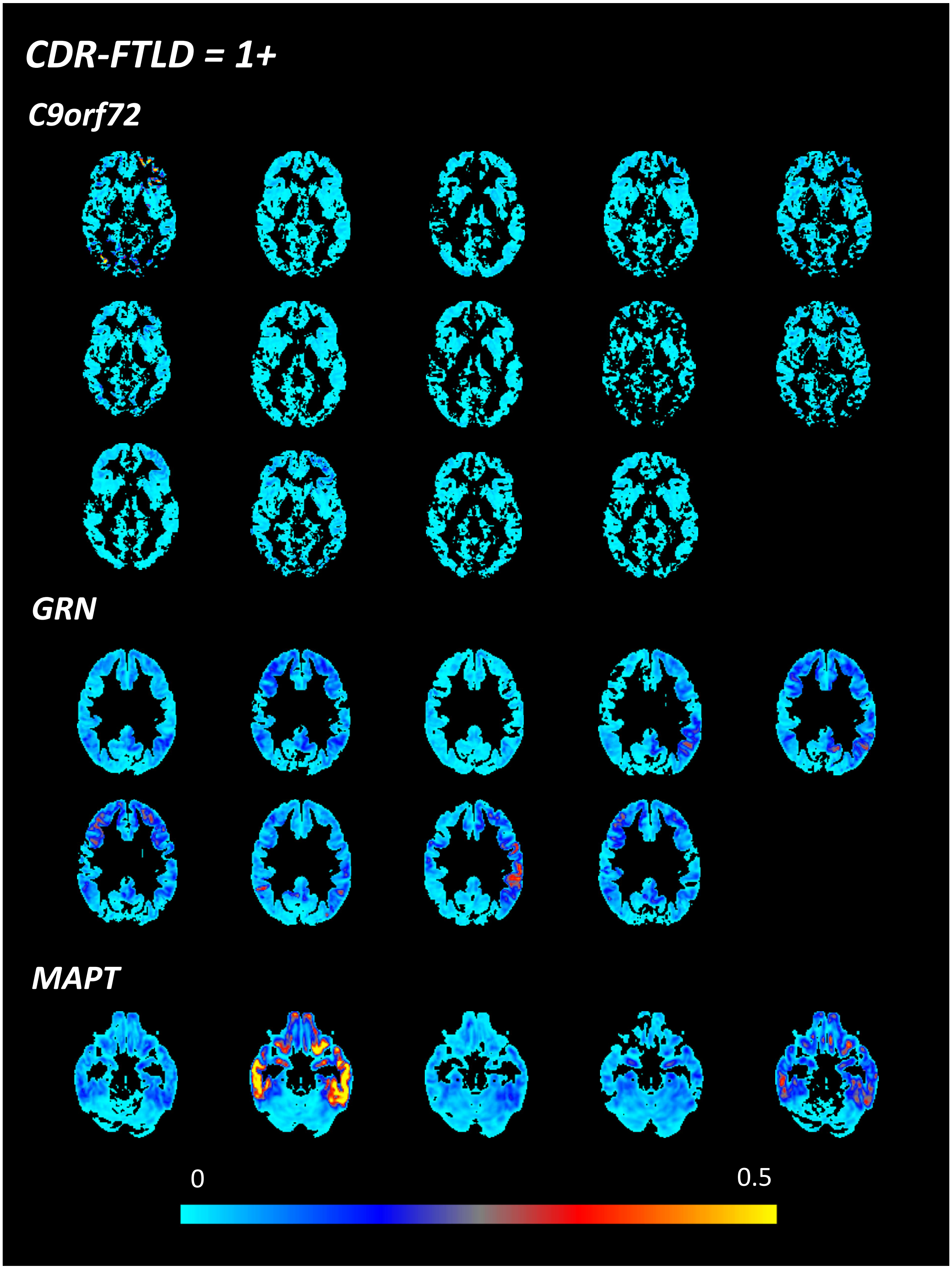

© 2020 Staffaroni AM et al. *JAMA Network Open*.

eFigure 7. Cross-Sectional Atrophy Maps in Each Genetic Group at Three Levels of Disease Severity

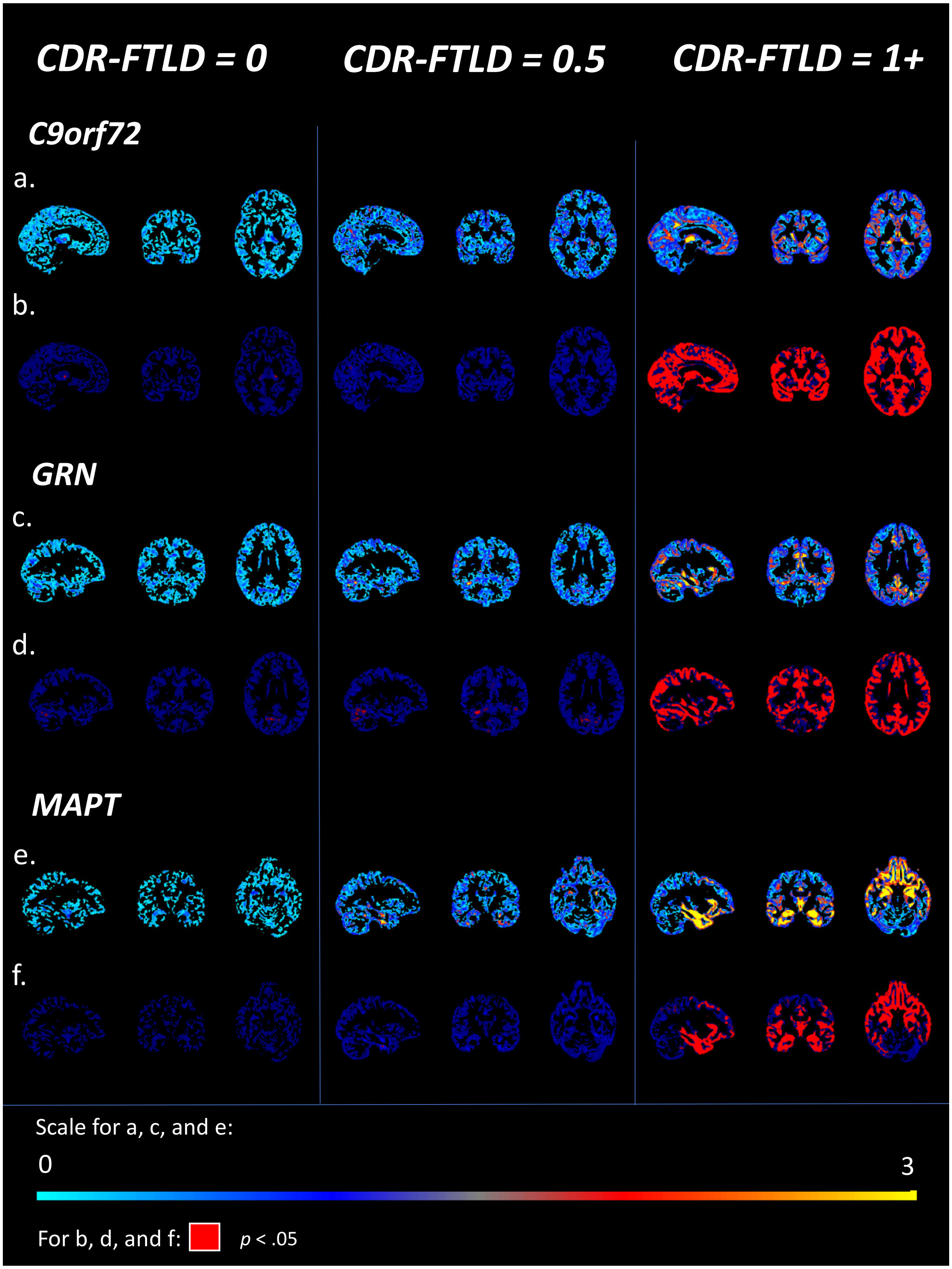

© 2020 Staffaroni AM et al. *JAMA Network Open*.

eFigure 8. Cross-Sectional Atrophy by Region of Interest

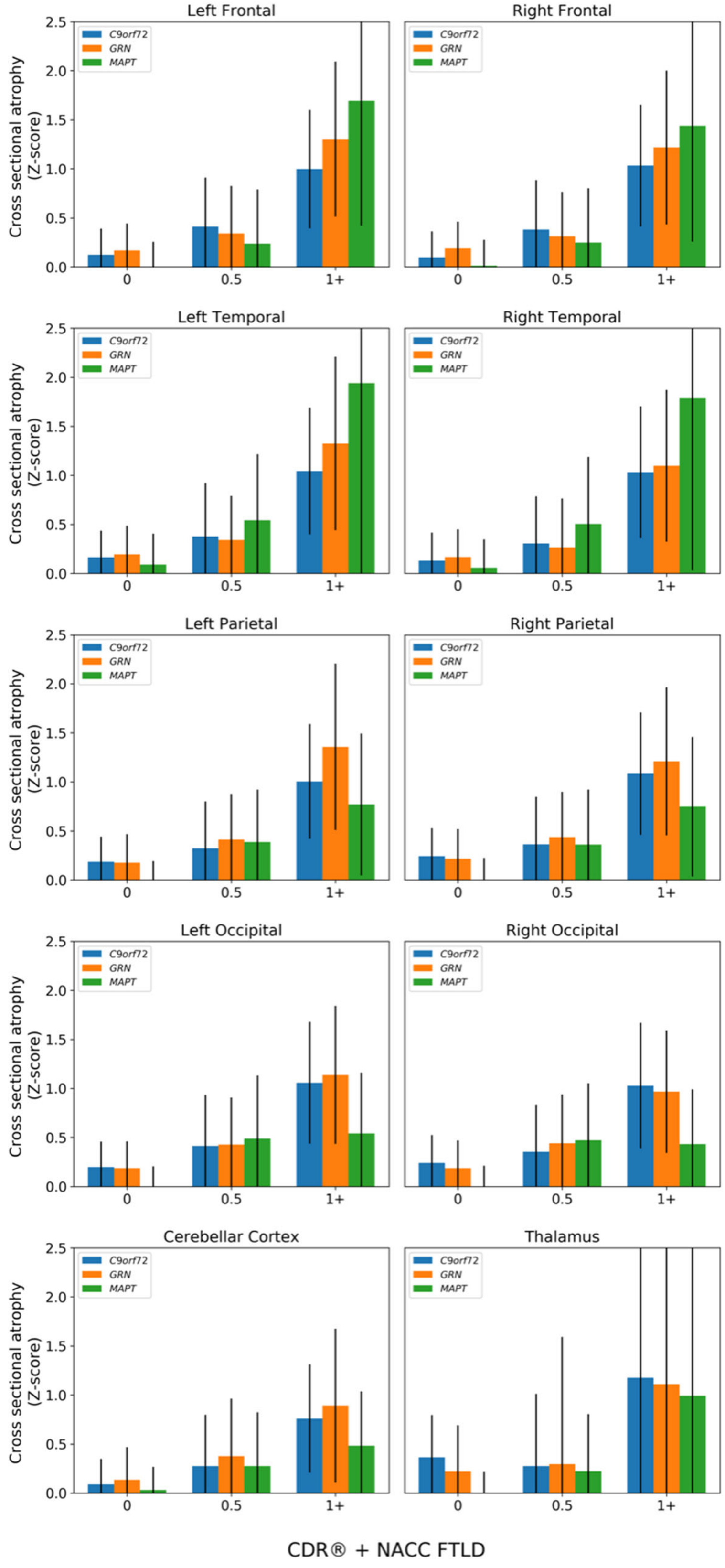

© 2020 Staffaroni AM et al. *JAMA Network Open*.

eTable 1. Demographic information for each subgroup

Group	CDR® + NACC FTLD	Mean Days Between Scans (SD)	Mean Number of Scans Per Subject (SD)	Baseline Age Mean (SD)*	Baseline Age Range*	Sex (% male)
**Controls**	-	443.01 (228.82)	2.30 (0.50)	47.17 (12.07)	24 – 76	43
**C9**	0	489.12 (310.02)	2.65 (0.75)	43.46 (11.83)	19 – 68	32
	0.5	403.57 (40.13)	2.40 (0.55)	59.90 (11.39)	50 – 75	42
	1+	391.32 (255.23)	2.57 (0.94)	57.66 (9.26)	33 – 67	50
**GRN**	0	370.96 (143.02)	2.33 (0.59)	53.22 (15.89)	22 – 71	53
	0.5	279.75 (172.58)	2.33 (0.52)	54.37 (10.74)	43 – 71	50
	1+	258.38 (180.16)	2.78 (1.09)	64.18 (5.67)	51 – 70	8
**MAPT**	0	480.64 (186.73)	2.47 (0.61)	39.41 (10.48)	22 – 57	61
	0.5	415.80 (37.63)	2.25 (0.5)	49.01 (10.11)	36 – 60	78
	1+	350.14 (52.10)	2.4 (0.55)	50.87 (11.95)	37 – 67	58

© 2020 Staffaroni AM et al. *JAMA Network Open*.

eTable 2. Diagnostic composition

Baseline Clinical Diagnosis (n)	Controls	CDR 0			CDR 0.5			CDR 1+			
		C9orf72	*GRN*	*MAPT*	*C9orf72*	*GRN*	*MAPT*	*C9orf72*	*GRN*	*MAPT*	
Clinically Normal	56	19	18	19		2					
Alcohol Abuse/Dependence		1									
Alzheimer's Disease							1	1	2		
ALS					1						
CBS (typical or variant)						1			1		
Behavioral Variant FTD							1	4	1	5	
FTD – Frontal								7	1		
FTD + ALS								1			
MCI – Language									1		
MCI – Mixed or Unspecified						1			1		
MCI – Psychiatric								1			
MCI – Behavior					1	1	2				
MCI – aMCIsd, aMCImd, naMCIsd, naMCImd					3						
PPA –nonfluent variant									1		
PPA – lopogenic variant									1		
Primary Psychiatric disorder – Mood	4										
**Total**	**60**	**20**	**18**	**19**	**5**	**6**	**4**	**14**	**9**	**5**	

Note. ALS = Amyotrophic lateral sclerosis; CBS = corticobasal syndrome; FTD = frontotemporal dementia; MCI = mild cognitive impairment; aMCI = amnestic MCI; na MCI = nonamnestic MCI; MCIsd = MCI single domain; MCImd = MCI multiple domain;

© 2020 Staffaroni AM et al. *JAMA Network Open*.

eTable 3. Distribution of Scanner Vendors by Genetic Group

Group	Scanner							
	Achieva	Biograph mMR	Intera	TrioTim	Discovery MR750	Prisma Fit	Skyra	Total
**Controls**	15	4	18	12	42	37	10	138
**C9**	18	0	11	33	17	19	3	101
**GRN**	0	2	9	36	20	8	6	81
**MAPT**	5	6	0	4	26	20	7	68

© 2020 Staffaroni AM et al. *JAMA Network Open*.

eTable 4. Mean and standard deviation (SD) of annualized rate of volume loss

CDR® + NACC FTLD	Left Frontal				Right Frontal			
	**Controls**	***C9orf72***	***GRN***	***MAPT***	**Controls**	***C9orf72***	***GRN***	***MAPT***
0	-170.2 (12.2)	-219.5 (74.7)	-234.9 (51)	-258.5 (98.8)	-160.3 (14.6)	-271.9 (118.4)	-266.9 (80.9)	-276.7 (118.7)
0.5		-392.8 (151.3)	-276.9(29.6)	-543.9 (300.6)		-309.7 (189.2)	-181.8 (89.8)	-576.4 (276.2)
1+		-285.2 (198.8)	-1530.3 (387.6)	-2269 (1574.1)		-250.8 (144.5)	-1169.2 (555)	-2052.8 (2005.7)
			**Left Temporal**				**Right Temporal**	
	**Controls**	***C9orf72***	***GRN***	***MAPT***	**Controls**	***C9orf72***	***GRN***	***MAPT***
0	-77.1 (13.1)	-128.9 (66.1)	-108.7 (17.7)	-231 (47.4)	-72.5 (16.8)	-104.9 (42.8)	-108.8 (25.5)	-149.6 (35.6)
0.5		-137.8 (14.6)	-126.8 (35.2)	-381 (207.9)		-42.9 (27.4)	-101.2 (17.2)	-315.4 (200.8)
1+		-76.8 (43.6)	-867.1 (308.4)	-1484.6 (1024.8)		-64.3 (46.3)	-433.3 (119.2)	-1163.9 (882.4)
			**Left Parietal**				**Right Parietal**	
	**Controls**	***C9orf72***	***GRN***	***MAPT***	**Controls**	***C9orf72***	***GRN***	***MAPT***
0	-104.9 (13.7)	-165.4 (104.7)	-140.3 (11.9)	-139.1 (26.5)	-102.2 (16)	-183.8 (157.1)	-181.5 (17.2)	-133.7 (40.9)
0.5		-224 (88.9)	-154.2 (70.9)	-303 (151.2)		-84.2 (91.2)	-119.6 (34.5)	-330.7 (123.2)
1+		-122.3 (156.8)	-896.4 (217)	-943.7 (650.6)		-123.6 (160)	-483.9 (108.2)	-729.5 (507.1)
			**Left Occipital**				**Right Occipital**	
	**Controls**	***C9orf72***	***GRN***	***MAPT***	**Controls**	***C9orf72***	***GRN***	***MAPT***
0	-27.9 (8.6)	-57.3 (26.3)	-36 (3.9)	-38.3 (10)	-31.4 (7.1)	-52.3 (14.4)	-38.4 (5.2)	-37.5 (8.7)
0.5		-28 (30.1)	-49.1 (14.5)	-110.2 (68.4)		14.8 (11.7)	-38.5 (36.1)	-98.7 (59.6)
1+		-3.6 (29.8)	-56.5 (43.6)	-82 (71.2)		-6.3 (38.2)	-55.7 (30)	-38.1 (88.4)
			**Thalamus**				**Cerebellum**	
	**Controls**	***C9orf72***	***GRN***	***MAPT***	**Controls**	***C9orf72***	***GRN***	***MAPT***
0	-10.5 (1.5)	-11 (4.8)	-13.4 (1.5)	-12.8 (12.9)	-76.2 (25.4)	-80.3 (28.4)	-74.4 (23.3)	-176.4 (21.3)
0.5		-31.3 (9.9)	-27.5 (3.9)	-77.5 (49.9)		-106.8 (184)	-53.6 (123.4)	-503.1 (736.8)
1+		-4.6 (9.7)	-45.1 (21.7)	-9.1 (71.8)		-79.5 (88.5)	-493.8 (50.8)	-652 (413.1)

© 2020 Staffaroni AM et al. *JAMA Network Open*.

**CDR® + NACC FTLD**	**FTLD-CDR SB**							
	**Controls**	***C9orf72***	***GRN***	***MAPT***				
0		0.1 (.01)	0.1 (.01)	0.1 (.01)				
0.5		0.4 (.1)	0.3 (.2)	0.3 (.1)				
1+		1.5 (.3)	1.4 (.5)	2.2 (1.0)				

Note. Coefficients represent the estimated, yearly rate of atrophy (mm^3)
Means and standard deviations were calculated from shrunk estimates derived from a linear mixed effects models
Because these estimates are shrunk towards the group mean, they include bias and should be interpreted with caution
Formal statistical tests were not performed due to this bias, but the means and SDs still provide important information about the relative effects of gene and disease severity

© 2020 Staffaroni AM et al. *JAMA Network Open*.

eTable 5. Cross-sectional volume by region of interest

CDR® + NACC FTLD		Left Frontal				Right Frontal		
	Controls	*C9orf72*	*GRN*	*MAPT*	Controls	*C9orf72*	*GRN*	*MAPT*
0	16550.3 (789.9)	16204 (807.6)	16118.9 (910.7)	16640 (1014.8)	16538.8 (809)	16266.8 (791.2)	16055.3 (910)	16547 (1070.6)
0.5		15521.5 (540)	15684.8 (1028.6)	16016.9 (1397.9)		15544.6 (591.9)	15744.1 (849)	15954 (1242.2)
1+		14154 (1298.6)	13440.5 (2042)	12683.4 (2344.7)		14011.9 (1484.6)	13678.9 (2447.5)	13234.7 (2014.6)
		Left Temporal				Right Temporal		
	Controls	*C9orf72*	*GRN*	*MAPT*	Controls	*C9orf72*	*GRN*	*MAPT*
0	10962.8 (445.2)	10727.4 (400.3)	10689.6 (533.8)	10885.7 (470.8)	10555.3 (394.8)	10376.8 (317.2)	10348.1 (484.7)	10509.6 (385.6)
0.5		10476.3 (353.8)	10480.2 (577.5)	10259.7 (802.1)		10176.5 (230.5)	10234.7 (452)	10000.4 (531.7)
1+		9563.2 (788.4)	9154.6 (1037.5)	8651.7 (1090.2)		9335.9 (734.2)	9270.8 (1530.2)	8627.6 (1236.9)
		Left Parietal				Right Parietal		
	Controls	*C9orf72*	*GRN*	*MAPT*	Controls	*C9orf72*	*GRN*	*MAPT*
0	9643.9 (532.7)	9322.9 (562.7)	9348.8 (640.9)	9723.9 (653.5)	10004.4 (543.1)	9588 (548.7)	9644.5 (698.1)	10060.8 (610.1)
0.5		9108.8 (427.4)	8975.5 (781.6)	9004.5 (906.1)		9396.4 (392)	9291.3 (782.7)	9422.7 (732.7)
1+		8048.6 (622.3)	7520.5 (1212.8)	8434.9 (715.5)		8283.2 (724.6)	8121.4 (1550.8)	8812.1 (514.9)
		Left Occipital				Right Occipital		
	Controls	*C9orf72*	*GRN*	*MAPT*	Controls	*C9orf72*	*GRN*	*MAPT*
0	3741.3 (191.9)	3622.8 (185.6)	3636.5 (222.5)	3752.3 (224.2)	3963.8 (172.4)	3822.7 (197.9)	3852.5 (225)	3979.2 (206.5)
0.5		3508.6 (124)	3493.6 (327.6)	3466.8 (411.6)		3755.2 (82.9)	3710.4 (323.9)	3702.2 (355.3)
1+		3126.9 (256.4)	3097.7 (458.3)	3432 (190)		3365 (263.6)	3415 (554.6)	3731.6 (190.6)
		Thalamus				Cerebellum		
	Controls	*C9orf72*	*GRN*	*MAPT*	Controls	*C9orf72*	*GRN*	*MAPT*
0	1628.1 (71.9)	1578.9 (62.4)	1571.4 (105.1)	1630.5 (83)	24936.1 (788.6)	24699 (438.1)	24674 (732.2)	24962.4 (763.4)
0.5		1572.1 (50.7)	1553.7 (97)	1593.1 (60.3)		24312.1 (436.2)	24244.9 (795.2)	24351.7 (1260)
1+		1395.1 (131.7)	1424.5 (199)	1413.8 (93)		23632.5 (661.6)	23427.9 (1070.5)	24076.1 (537.6)

Note. Descriptive statistics are presented for each ROI in units of mm^3.
The last observation of each individual was included in these estimates
Carriers of the *C9orf72* repeat expansion show atrophy compared to controls at a similar level as carriers of *GRN* and *MAPT* mutation

© 2020 Staffaroni AM et al. *JAMA Network Open*.
